# A Nuclear Export Signal in KHNYN Required for Its Antiviral Activity Evolved as ZAP Emerged in Tetrapods

**DOI:** 10.1128/jvi.00872-22

**Published:** 2023-01-12

**Authors:** Maria J. Lista, Mattia Ficarelli, Harry Wilson, Dorota Kmiec, Rebecca L. Youle, Joseph Wanford, Helena Winstone, Charlotte Odendall, Ian A. Taylor, Stuart J. D. Neil, Chad M. Swanson

**Affiliations:** a King’s College London, Department of Infectious Diseases, London, United Kingdom; b The Francis Crick Institute, Macromolecular Structure Laboratory, London, United Kingdom; Icahn School of Medicine at Mount Sinai

**Keywords:** KHNYN, N4BP1, ZAP, PARP13, ZC3HAV1, antiviral protein evolution, HIV, CpG, innate immunity, interferon-stimulated gene

## Abstract

The zinc finger antiviral protein (ZAP) inhibits viral replication by directly binding CpG dinucleotides in cytoplasmic viral RNA to inhibit protein synthesis and target the RNA for degradation. ZAP evolved in tetrapods and there are clear orthologs in reptiles, birds, and mammals. When ZAP emerged, other proteins may have evolved to become cofactors for its antiviral activity. KHNYN is a putative endoribonuclease that is required for ZAP to restrict retroviruses. To determine its evolutionary path after ZAP emerged, we compared KHNYN orthologs in mammals and reptiles to those in fish, which do not encode ZAP. This identified residues in KHNYN that are highly conserved in species that encode ZAP, including several in the CUBAN domain. The CUBAN domain interacts with NEDD8 and Cullin-RING E3 ubiquitin ligases. Deletion of the CUBAN domain decreased KHNYN antiviral activity, increased protein expression and increased nuclear localization. However, mutation of residues required for the CUBAN domain-NEDD8 interaction increased KHNYN abundance but did not affect its antiviral activity or cytoplasmic localization, indicating that Cullin-mediated degradation may control its homeostasis and regulation of protein turnover is separable from its antiviral activity. By contrast, the C-terminal residues in the CUBAN domain form a CRM1-dependent nuclear export signal (NES) that is required for its antiviral activity. Deletion or mutation of the NES increased KHNYN nuclear localization and decreased its interaction with ZAP. The final 2 positions of this NES are not present in fish KHNYN orthologs and we hypothesize their evolution allowed KHNYN to act as a ZAP cofactor.

**IMPORTANCE** The interferon system is part of the innate immune response that inhibits viruses and other pathogens. This system emerged approximately 500 million years ago in early vertebrates. Since then, some genes have evolved to become antiviral interferon-stimulated genes (ISGs) while others evolved so their encoded protein could interact with proteins encoded by ISGs and contribute to their activity. However, this remains poorly characterized. ZAP is an ISG that arose during tetrapod evolution and inhibits viral replication. Because KHNYN interacts with ZAP and is required for its antiviral activity against retroviruses, we conducted an evolutionary analysis to determine how specific amino acids in KHNYN evolved after ZAP emerged. This identified a nuclear export signal that evolved in tetrapods and is required for KHNYN to traffic in the cell and interact with ZAP. Overall, specific residues in KHNYN evolved to allow it to act as a cofactor for ZAP antiviral activity.

## INTRODUCTION

Viral RNAs are targeted by diverse antiviral systems in eukaryotes to restrict viral replication. In plants and invertebrates, RNAi forms a major system to inhibit viral replication and this pathway was likely present in the last common ancestor of eukaryotes (1, 2). In vertebrates, the interferon system replaced RNAi as the predominant mechanism to inhibit viral replication, though RNAi is still active in some mammalian cell types, such as stem cells (1, 3). This protein-based system consists of pattern recognition receptors (PRRs) that bind pathogen-associated molecular patterns (PAMPs) (4). When a PRR binds a PAMP, signal transduction pathways are activated that induce transcription of antiviral cytokines, including interferons (IFNs) (5). The IFN system likely evolved in early vertebrates and is present in fishes and the tetrapod lineages (amphibians, reptiles, birds, and mammals) (6). When IFNs are produced by an infected cell, they signal in an autocrine and paracrine manner to activate the expression of interferon-stimulated genes (ISGs). These have diverse functions, and some are directly antiviral (7).

ZAP (also known as ZC3HAV1 or PARP13) is an ISG that evolved from PARP12, which is an ISG expressed throughout the vertebrate lineage (8–11). ZAP emerged during tetrapod evolution and there are clear orthologs in mammals, reptiles and birds that have antiviral activity (8). ZAP binds viral RNA containing CpG dinucleotides to target it for degradation and inhibit its translation (11–15). ZAP interacts with several cellular proteins to form the ZAP antiviral system (16–22), and the emergence of ZAP may have provided an opportunity for these proteins to evolve in specific ways to act as cofactors, though little is known in this regard.

KHNYN is a ZAP-interacting protein that is required for it to restrict retroviruses (22). Unlike ZAP, KHNYN is not a core mammalian ISG (9). It has 2 human paralogs, NYNRIN and N4BP1. NYNRIN evolved from a KHNYN gene duplication in which the RNase H and integrase domains from an endogenous retrovirus replaced the last exon of KHNYN and it has been suggested to play a role in the regulation of placental development (23, 24). N4BP1 is a predominantly nucleolar protein whose expression is induced by type I interferon (9, 25–28). The best characterized function for N4BP1 is modulating the NF-KB pathway and it has also been proposed to regulate HIV-1 gene expression and the E3 ubiquitin ligase Itch (29–33). N4BP1 also has a genetic interaction with ZAP in that ZAP is required for N4BP1 antiviral activity (34).

Because it is unclear how KHNYN evolved to act as a ZAP cofactor, we characterized how specific changes in KHNYN that appeared after ZAP emerged in tetrapods regulate its antiviral activity. In mammalian and reptile species with clear ZAP orthologs, KHNYN contains a CRM1 nuclear export signal (NES) in its C-terminal CUBAN domain. This NES is required for KHNYN to traffic from the nucleus to the cytoplasm to interact with ZAP and mediate its antiviral activity. The penultimate and C-terminal positions of this NES evolved in the tetrapod lineage coincident with the emergence of ZAP.

## RESULTS

### Identification of residues in KHNYN that evolved coincident with the emergence of ZAP.

**FIG 1:**
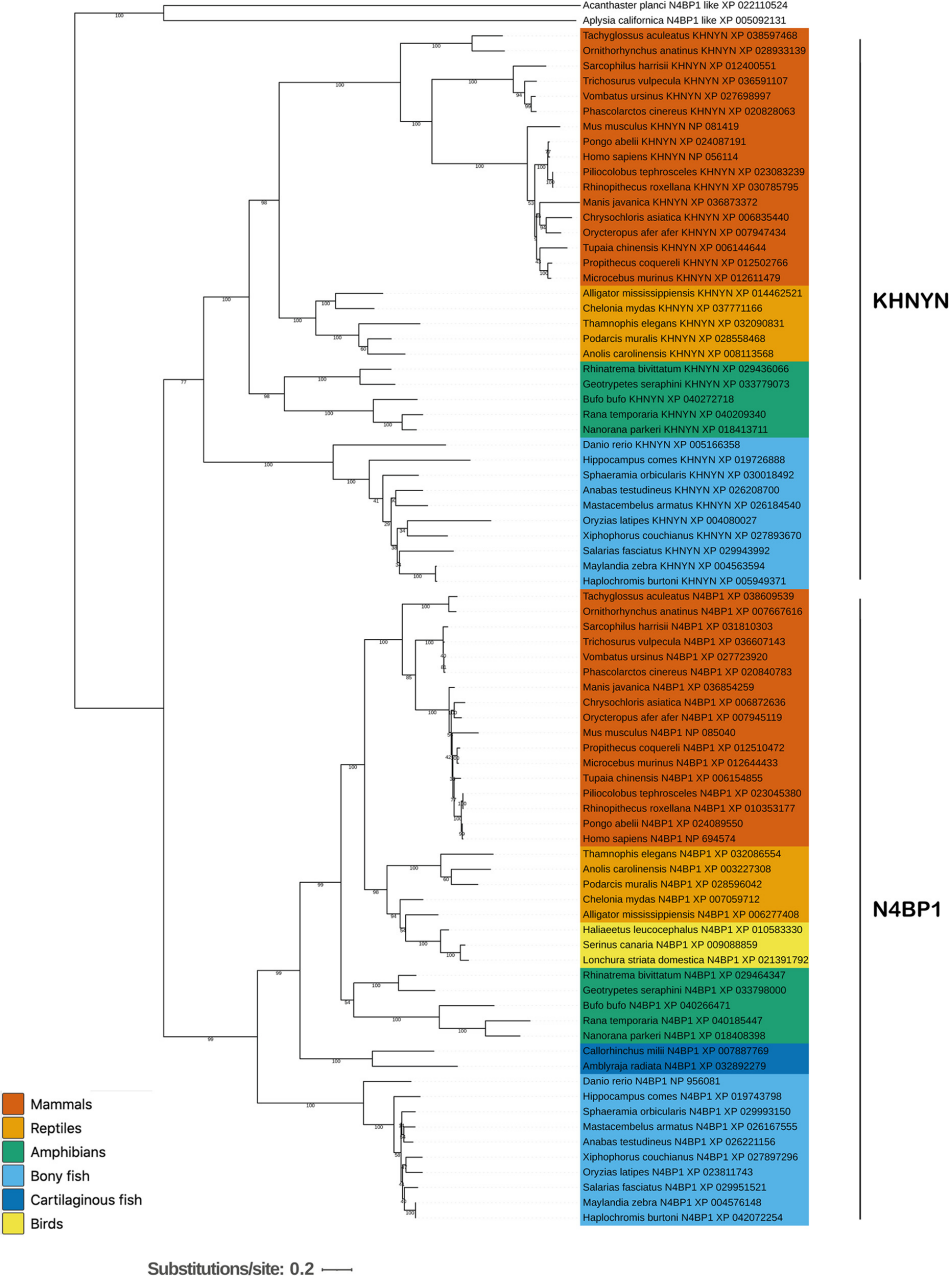
KHNYN and N4BP1 are paralogs that evolved from a gene duplication potentially in the bony vertebrate (Euteleostomi) lineage. Maximum likelihood phylogenetic tree of KHNYN and N4BP1 amino acid sequences. Representative sequences from mammals, reptiles, birds, amphibians, and fishes were aligned and a maximum likelihood phylogeny was inferred with RAxML using the LG substitution model. The crown-of-thorns starfish and Californian sea hare were used as outgroups to root the tree. The scalebar is the amino acid substitutions per site.

To understand how KHNYN evolved to function as a ZAP cofactor, we first identified N4BP1 and KHNYN orthologs in invertebrates and vertebrates. While the complete N4BP1-KHNYN evolutionary pathway is currently unclear, N4BP1-like proteins are present in invertebrates such as echinoderms (e.g., crown-of-thorns starfish [*Acanthaster planci*]) and mollusks (e.g., California sea hare [*Aplysia californica*]) (Fig. 1). These animals have one N4BP1-like protein. Within chordates, N4BP1-like proteins are found in lancelets and throughout vertebrates. A single N4BP1-like protein is found in cartilaginous fishes, while bony fishes, amphibians, reptiles, and mammals have clear N4BP1 and KHNYN orthologs (Fig. 1). This indicates that a gene duplication event may have occurred early in the bony vertebrate lineage that led to N4BP1 and KHNYN paralogs. However, while N4BP1 orthologs are present in birds, KHNYN orthologs are not, suggesting that it has been lost in this lineage. NYNRIN originated as a fusion between KHNYN and a Ty3/Gypsy *pol* gene in the therian stem lineage and is present in most marsupials and placental mammals (24). Because it emerged much later than ZAP in tetrapod evolution, we have not considered it further in this study.

**FIG 2:**
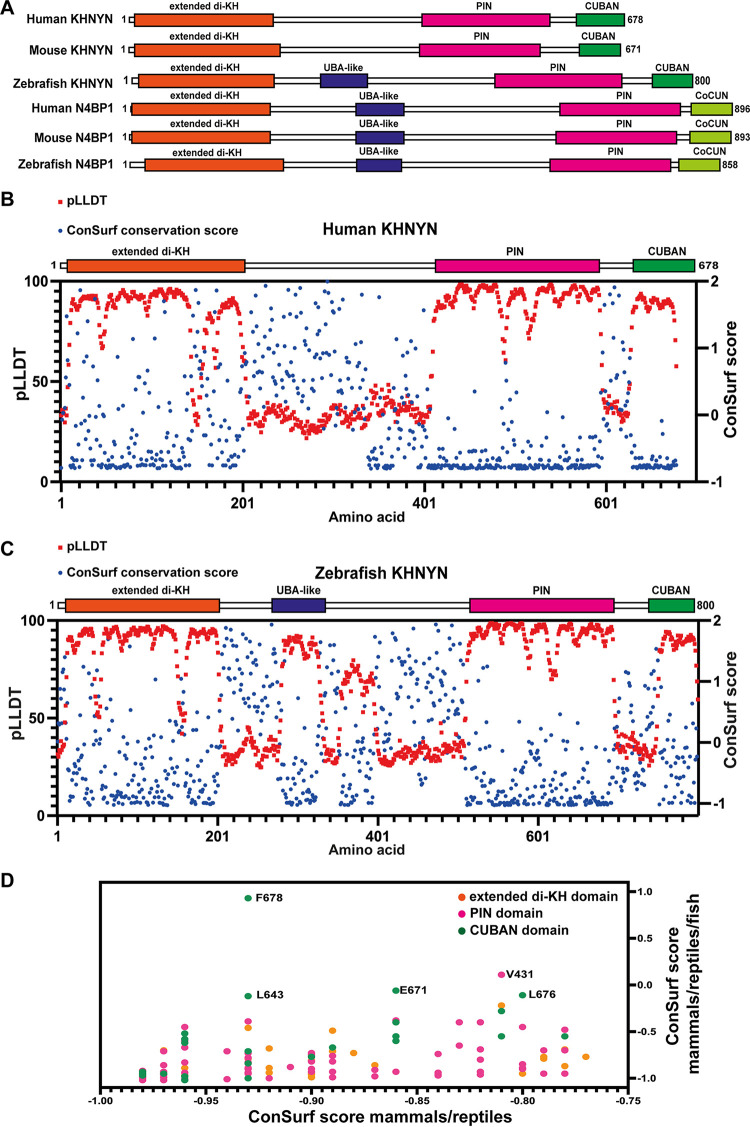
Some residues that are conserved in mammalian and reptile KHNYN orthologs are not conserved in bony fish orthologs. (A) Schematic of the extended di-KH, UBA-like, PIN and CUBAN/CoCUN domains in human, mouse, and zebrafish KHNYN and N4BP1. (B) KHNYN orthologs in placental mammals have three conserved domains. The AlphaFold structure confidence score (pLLDT) for human KHNYN and the ConSurf conservation score from a MSA of placental mammal KHNYN protein sequences were plotted for each amino acid in the human KHNYN sequence. A schematic showing the extended di-KH domain, PIN domain and CUBAN domain in KHNYN is shown above the plot. (C) The AlphaFold structure confidence score (pLLDT) for zebrafish KHNYN and the ConSurf conservation score from a multiple sequence alignment of bony fish KHNYN protein sequences were plotted for each amino acid in the zebrafish KHNYN sequence. (D) Specific residues in KHNYN are less conserved in the mammal/reptile/bony fish MSA than the mammal/reptile MSA. The ConSurf conservation score for the mammal/reptile MSA (*x* axis) was plotted against the ConSurf conservation score for the mammal/reptile/bony fish MSA (*y* axis) for each residue in human KHNYN. Residues that substantially changed are labeled. Residues in the extended di-KH domain are colored orange, residues in the PIN domain are colored pink, and residues in the CUBAN domain are colored green.

Because KHNYN had no known function before it was identified as a ZAP cofactor (22), it remains poorly characterized and little is known about how its overall domain structure contributes to its antiviral activity. In contrast, N4BP1 functional studies have defined how its domains are required for at least some of its activities (25, 27, 30, 32, 35). Since N4BP1 is a KHNYN paralog, most of these domains are conserved between the proteins and may provide insight into KHNYN evolution and activity. N4BP1 has been reported to contain four structural domains: two N-terminal K homology (KH) domains in close proximity to form a di-KH domain, an ubiquitin-associated (UBA)-like domain, a PilT N-terminal (PIN) nuclease domain and a C-terminal Cousin of CUBAN (CoCUN) domain (25, 27, 30, 32, 35). Inspection of the AlphaFold models for human, mouse, and zebrafish N4BP1 also supports the presence of these 4 structural domains (Fig. 2A, Fig. S1) (36, 37). The AlphaFold N4BP1 di-KH domain has three additional alpha helices at its C-terminus which is similar to a currently unpublished crystal structure for this domain (PDB 6q3v), and we refer to this as an extended di-KH domain (16). We then inspected the AlphaFold models for human and mouse KHNYN to determine its domain structure. These models predict 3 structural domains that are similar to N4BP1: an extended di-KH domain, a PIN domain, and a C-terminal cullin-binding domain associating with NEDD8 (CUBAN) domain (Fig. 2A and Fig. S1, S2A, and S2C) (16, 27, 35–38). It should be noted that the AlphaFold model for KHNYN needs to be experimentally validated and, of these 3 domains, only the CUBAN domain has been structurally characterized with 3 alpha helices present from residues 632 to 678 (38).

To determine if the AlphaFold model for full-length KHNYN is supported by evolutionary conservation in mammalian KHNYN sequences, we used ConSurf (39, 40) to determine the normalized conservation score of each amino acid in a multiple sequence alignment (MSA) of placental mammals (Data set S1) and plotted it against the structural confidence score (pLDDT) in the AlphaFold model of human KHNYN (Fig. 2B). Of note, the ConSurf conservation score corresponds to the evolutionary rate of the residue and lower numbers indicate more conserved residues. This analysis supports the three-domain model for mammalian KHNYN in that the regions with high AlphaFold confidence scores are well conserved evolutionarily, though an unstructured region N-terminal to the PIN domain also appears to be conserved (Fig. 2B and Data set S1). The function of the extended di-KH domain in KHNYN is unknown but a deletion in this domain reduced its antiviral activity (22). The PIN domain in KHNYN is a putative endoribonuclease domain and mutation of potential catalytic residues in this domain inhibits its antiviral activity (22). The CUBAN domain binds NEDD8 and ubiquitin, both of which are members of the ubiquitin-like family, and preferentially binds monomeric NEDD8 over ubiquitin (38). NEDD8 binding mediates an interaction between KHNYN and neddylated cullin–RING E3 ubiquitin ligases (38). However, the role of the CUBAN domain for KHNYN antiviral activity is not known.

The AlphaFold model for zebrafish KHNYN has 5 potential domains which are supported by evolutionary conservation in the bony fish lineage, including the extended di-KH domain, the PIN domain and the CUBAN domain (Fig. 2C and Data set S1). One of the additional domains aligns with the UBA-like domain in N4BP1 and is not conserved in mammalian KHNYN orthologs (Fig. 2C and Fig. S1, S2A to C) (32). The other potential domain has lower structure confidence scores than the other domains, does not appear to be present in mammalian KHNYN orthologs and the function of this region is unknown (Fig. 2C and Fig. S1). Overall, it appears that N4BP1 has 4 domains and these are conserved in bony fish KHNYN (Fig. 2A), indicating that this is the primordial domain structure for these proteins in vertebrates. The UBA-like domain has been lost in mammalian KHNYN orthologs and they have three clear structural domains.

Bony vertebrates (Euteleostomi) are divided into sarcopterygians (lobe-finned fish, which include tetrapods) and actinopterygians (ray-finned fish) and these lineages diverged from each other ~ 450 million years ago (41, 42). Because there is considerable evolutionary distance between these clades, and ray-finned fish do not encode known ZAP orthologs (8), we hypothesized that comparing MSAs of KHNYN orthologs in tetrapods to ray-finned fishes may highlight residues that are required for KHNYN to function as a ZAP cofactor. We used ConSurf to compare a MSA of mammal and reptile KHNYN orthologs to a MSA of mammal, reptile, and ray-finned fish KHNYN orthologs. ConSurf divides the conservation scores into9 categories. We used the residues in the category with the highest conservation (category 9, Data set S1) in the mammal and reptile orthologs to compare the conservation scores for each residue in human KHNYN for the mammal/reptile MSA to the score in the mammal/reptile/bony fish MSA (Fig. 2D). This identified 5 residues (V431, L643, E671, L676, and F678) in which the conservation score substantially increased when bony fish were included in the MSA, indicating that these residues are not conserved in this clade. Surprisingly, even though the CUBAN domain is the smallest domain in KHNYN, 4 of these 5 residues are located in this domain. Since there is an experimentally determined structure for the CUBAN domain (38), and its role for KHNYN antiviral activity is not known, we focused on this domain.

### The CUBAN domain in KHNYN regulates its abundance and subcellular localization.

**FIG 3:**
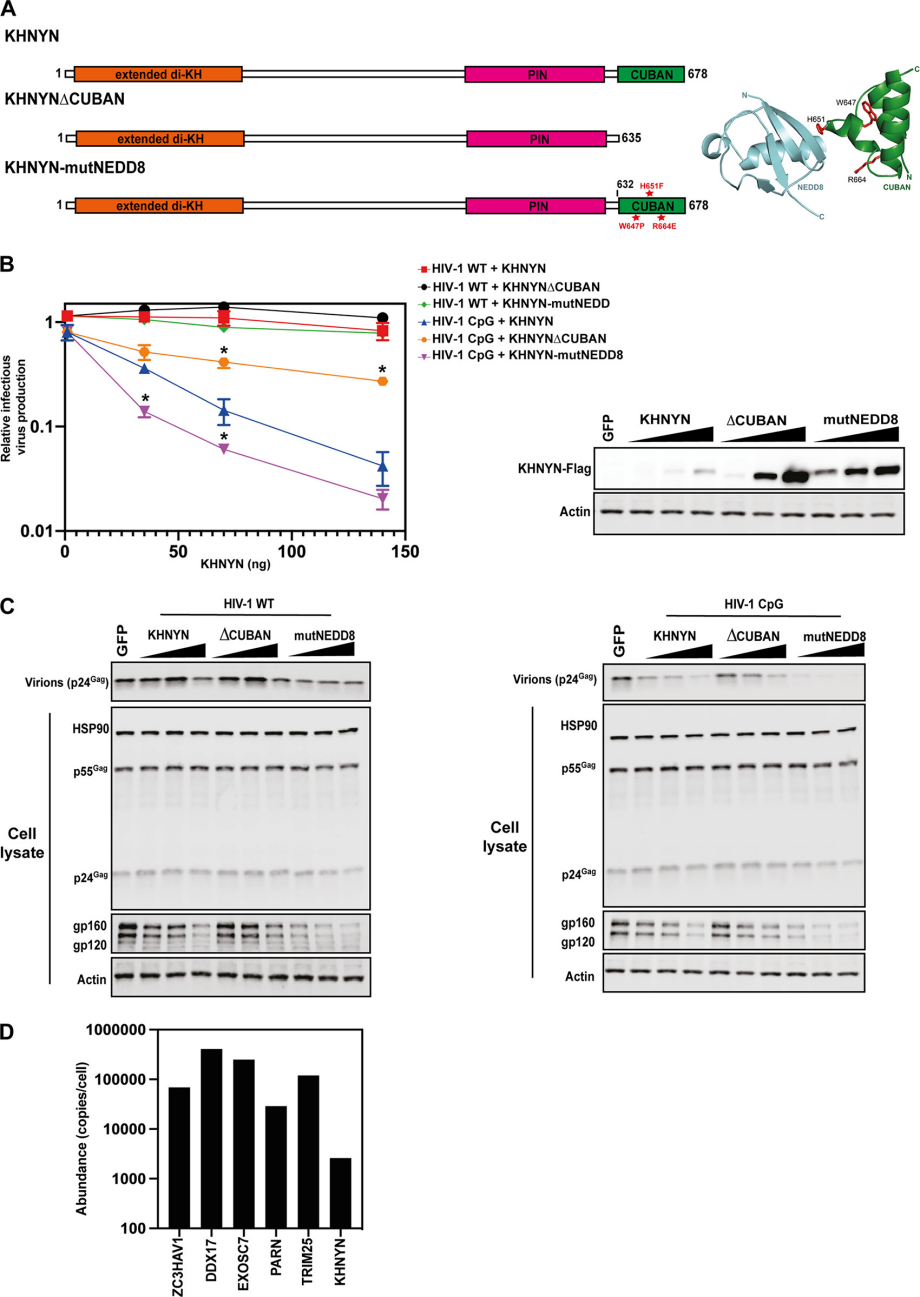
The CUBAN domain is essential for KHNHN antiviral activity and protein localization. (A) Left panel: Schematic representation of KHNYN, KHNYNΔ636-678 (KHNYNΔCUBAN) and KHNYN W647P/H651F/R664E (KHNYNmutNEDD8). The residues mutated in KHNYNmutNEDD8 are highlighted in red. Right panel: Cartoon representation of the NEDD8-CUBAN complex structure (PDB: 2N7K). The residues mutated in KHNYNmutNEDD8 are highlighted in stick representation in red. (B) Left panel: Infectious virus production from KHNYN CRISPR HeLa cells co-transfected with pHIV-1_WT_ or pHIV-1_CpG_ and increasing amount of CRISPR-resistant wild-type pKHNYN-Flag, pKHNYNΔCUBAN-Flag or pKHNYN-mutNEDD8-Flag plasmids. Each point shows the average value of three independent experiments normalized to the value obtained for pHIV-1_WT_ at 0 ng pKHNYN. *, *P* < 0.05 as determined by a two-tailed *unpaired t test* comparing wild-type KHNYN and the mutant KHNYN at each concentration in the HIV-1_CpG_ samples. Right panel: representative Western blot of the protein level of wild-type KHNYN-FLAG, KHNYNΔCUBAN-FLAG and KHNYNmutNEDD8-FLAG corresponding to the titration shown in the left panel. (C) Representative Western blots for Gag and Env in cell lysates as well as virion production in the media. (D) Expression of ZAP cofactors that regulate viral RNA degradation in HeLa cells. Data is from reference (50).

The CUBAN domain in KHNYN has been shown to bind to NEDD8 and mediate an interaction between KHNYN and neddylated cullin-RING E3 ubiquitin ligases, including CUL1, CUL2, CUL3, and CUL4 (38). However, it is not known whether the CUBAN domain is required for antiviral activity. HIV type 1 (HIV-1) is a common model system to study the antiviral activity of ZAP and its cofactors (8, 15, 22, 43–45). While HIV-1 is highly depleted in CpG dinucleotides, which makes it poorly targeted by ZAP, when a specific region in HIV-1 *env* is engineered to contain additional CpGs through synonymous mutations (HIV-1_CpG_), the virus becomes ZAP-sensitive (15, 46–49). This allows matched ZAP-resistant and ZAP-sensitive viruses to be tested to characterize how ZAP and its cofactors restrict viral replication. To determine whether the CUBAN domain is required for KHNYN antiviral activity, we co-transfected CRISPR-resistant wild-type pKHNYN or pKHNYN with a CUBAN domain deletion (KHNYNΔCUBAN, Fig. 3A) and either pHIV-1_WT_ or pHIV-1_CpG_ into KHNYN CRISPR HeLa cells. Deletion of the CUBAN domain led to a large decrease in KHNYN antiviral activity on HIV-1_CpG_ infectious virus production, even though KHNYNΔCUBAN was expressed at much higher levels than the wild-type protein (Fig. 3B and C). This indicates that the CUBAN domain may be required for both KHNYN homeostatic turnover and antiviral activity.

The CUBAN domain comprises a three-helix bundle (α1 = T632-R640, α2 = K652-L657, α3 = Y662-F678) connected by 2 loops (38). The binding interface between the CUBAN domain and NEDD8 is formed from a negatively charged motif in NEDD8 and a positively charged surface in the CUBAN α2 and surrounding residues. Quantitative proteomics in HeLa cells have shown that many of the ZAP cofactors required for viral RNA degradation are expressed at much higher levels than KHNYN (Fig. 3D) and there are ~ 25-fold more ZAP molecules/cell than KHNYN (50). Therefore, the interaction between the CUBAN domain and NEDD8 could contribute to the low expression level of endogenous KHNYN (50). Three mutations have previously been shown to decrease binding of the CUBAN domain to NEDD8: W647P, H651F, and R664E (Fig. 3A) (38). When these mutations were introduced in KHNYN (KHNYN-mutNEDD8), they increased its protein abundance and moderately increased its antiviral activity for HIV-1_CpG_ (Fig. 3B and C). This suggests that the CUBAN domain-NEDD8 interaction likely regulates KHNYN turnover but not antiviral activity and indicates that there may be another functional motif in the CUBAN domain.

**FIG 4:**
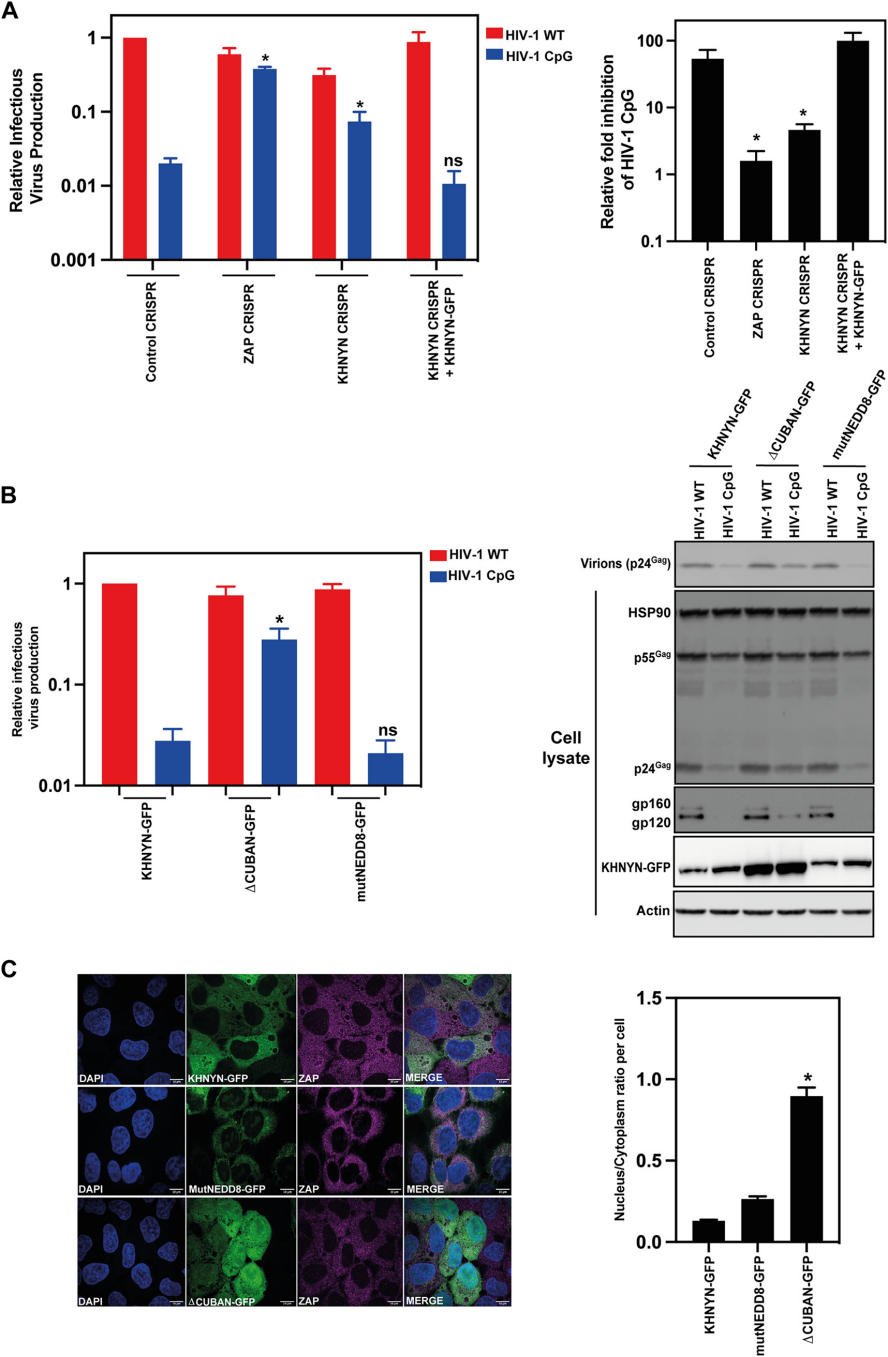
Deletion of the CUBAN domain re-localizes KHNYN to the nucleus. (A) Left panel: Control CRISPR, ZAP CRISPR, KHNYN CRISPR or KHNYN CRISPR + KHNYN-GFP cells were infected with VSV-G pseudotyped HIV-1_WT_ or HIV-1_CpG_. 48 h postinfection, the cell supernatant was harvested and infectious virus production was measured in TZM-bl cells. Each bar shows the average value of three independent experiments normalized to the value obtained for HIV-1_WT_ in control CRISPR cells. *, *P* < 0.05 as determined by a two-tailed *unpaired t test* comparing HIV-1_CpG_ in each cell line to the control CRISPR cell line. Right panel: The relative fold inhibition for HIV-1_CpG_ relative to HIV-1_WT_. *, *P* < 0.05 as determined by a two-tailed *unpaired t test* comparing each cell line to the control CRISPR cell line. (B) Left panel: Infectious virus production from HeLa KHNYN CRISPR cells stably expressing wild-type KHNYN-GFP, KHNYNΔCUBAN-GFP or KHNYNmutNEDD8-GFP. All cell lines were transfected with HIV-1_WT_ or HIV-1_CpG_. Each bar shows the average value of five independent experiments normalized to the value obtained for wild-type KHNYN co-transfected with pHIV-1_WT_. *, *P* < 0.05 as determined by a two-tailed *unpaired t test* comparing wild-type KHNYN and the mutant KHNYN construct in the HIV-1_CpG_ samples. Data are represented as mean ± SD. Right panel: Representative Western blots for Gag, Env and GFP in cell lysates as well as virion production in the media. (C) Left panel: Confocal microscopy images of the KHNYN-GFP cell lines (green) co-stained with endogenous ZAP (magenta), scale bar is 10 μm. Right panel: Signal quantification per cell (50 cells total per condition) of the ratio of KHNYN nuclear and cytoplasmic distribution in the KHNYN-GFP cell lines. *, *P* < 0.05 as determined by a two-tailed *unpaired t test* comparing the nuclear/cytoplasmic ratio between each sample.

To analyze the subcellular localization of the mutant KHNYN proteins, KHNYN-GFP, KHNYN-mutNEDD8-GFP, and KHNYNΔCUBAN-GFP were stably expressed in the KHNYN CRISPR HeLa cells. As expected, the KHNYN-GFP cells restricted HIV-1_CpG_ (Fig. 4A). To confirm the effect of the CUBAN domain mutations in the context of the KHNYN-GFP stable cell lines, these cells were transfected with pHIV-1_WT_ or pHIV-1_CpG_. Similar to the transient transfection experiments described above (Fig. 3B and C), deletion of the CUBAN domain decreased KHNYN-GFP antiviral activity while introducing the mutations that reduce NEDD8 binding did not affect it (Fig. 4B). Of note, the increase in KHNYN abundance due to the CUBAN domain deletion or mutations that decrease NEDD8 binding are less pronounced in the KHNYN-GFP cell lines than in the experiments with transiently transfected KHNYN-FLAG constructs (Fig. 3B and 4B), possibly because the GFP fusion stabilizes the wild-type protein. Interestingly, while KHNYN-mutNEDD8-GFP localized to the cytoplasm similar to wild-type KHNYN-GFP, KHNYNΔCUBAN-GFP had a substantial increase in nuclear localization (Fig. 4C). Therefore, the CUBAN domain regulates KHNYN subcellular localization in addition to its homeostatic turnover and antiviral activity.

### KHNYN has a nuclear export signal at the C-terminus of the CUBAN domain that is required for antiviral activity.

**FIG 5:**
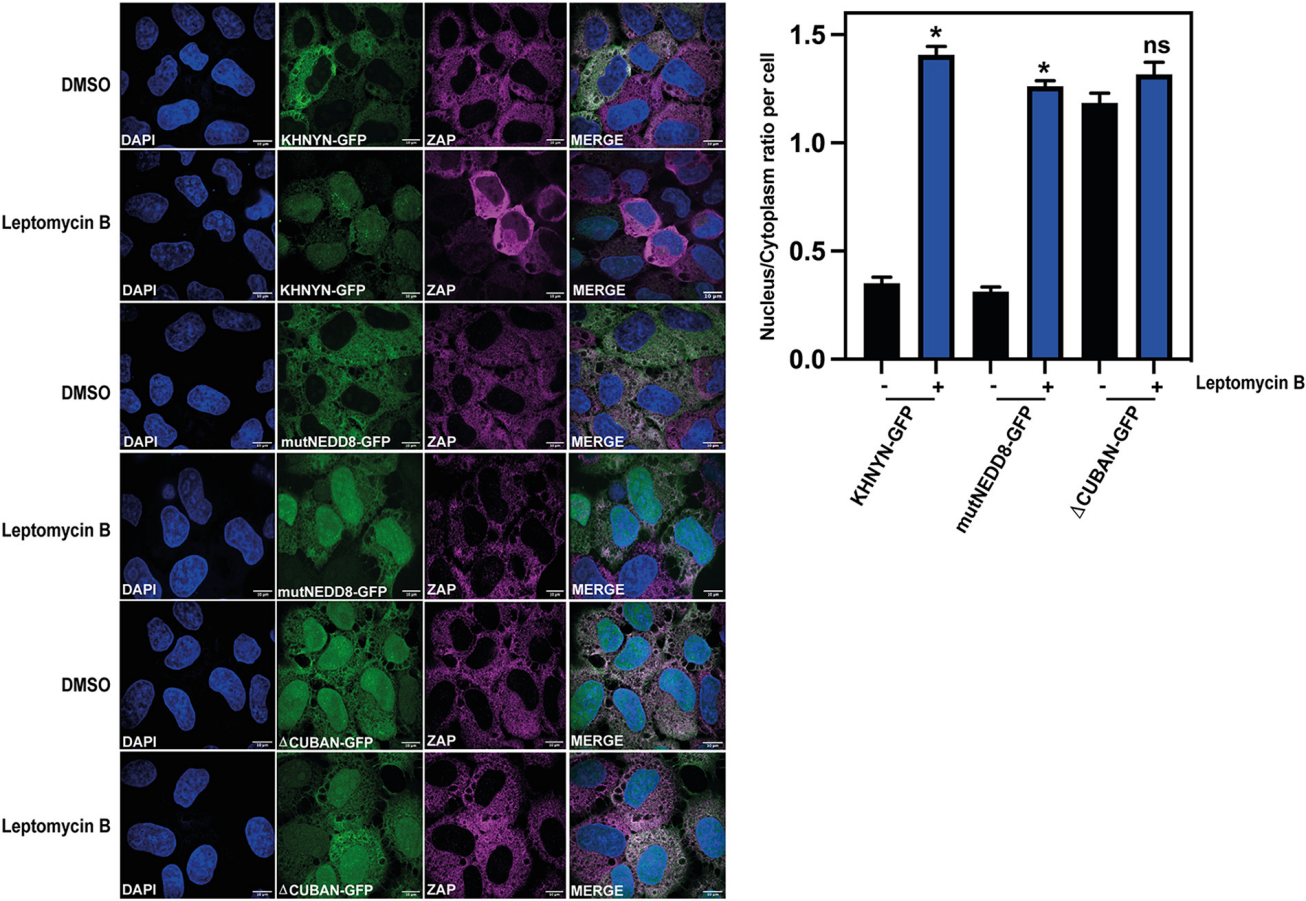
CRM1 inhibition by Leptomycin B re-localizes KHNYN to the nucleus. Left panel: Representative confocal microscopy images of KHNYN CRISPR HeLa cells stably expressing either wild-type KHNYN-GFP or the indicated mutants before and after a 4-h treatment with 50 nM Leptomycin B. KHNYN-GFP is shown in green, endogenous ZAP co-staining is shown in magenta, scale bar is 10 μm. Right panel: Signal quantification per cell (50 cells in total per condition) of the ratio of KHNYN nuclear and cytoplasmic distribution for KHNYN-GFP or the indicated mutant protein before and after Leptomycin B treatment. *, *P* < 0.05 as determined by a two-tailed unpaired t test comparing the nuclear/cytoplasmic ratio between each sample.

CRM1 (also known as XPO1) is a nuclear export protein that mediates trafficking of many cellular proteins and ribonucleoprotein complexes from the nucleus to the cytoplasm (51). CRM1 binds leucine-rich nuclear export signals (NESs) in cargo proteins and the binding groove for the NES is highly conserved from yeast to human orthologs (51–56). Because KHNYN has previously been identified as a CRM1 cargo protein in a large-scale proteomics screen (57), we confirmed that KHNYN uses the CRM1 nuclear export pathway by analyzing the subcellular localization of KHNYN-GFP, KHNYN-mutNEDD8-GFP and KHNYNΔCUBAN-GFP in the absence and presence of leptomycin B, a small molecule inhibitor of CRM1 (58, 59). Addition of leptomycin B to the KHNYN-GFP stable cell lines substantially increased wild-type KHNYN-GFP or KHNYN-mutNEDD8-GFP nuclear localization to levels similar to KHNYNΔCUBAN-GFP (Fig. 5). However, the subcellular localization of KHNYNΔCUBAN-GFP was not affected by leptomycin B, indicating that the CRM1 NES is present in the CUBAN domain. While ZAP also has a CRM1 NES and has been reported to be a CRM1-dependent nucleocytoplasmic shuttling protein (57, 60), it was not substantially re-localized to the nucleus by leptomycin B treatment (Fig. 5). This suggests that, in these cells, ZAP is sequestered in the cytoplasm and is not undergoing CRM1-dependent nuclear-cytoplasmic trafficking.

**FIG 6:**
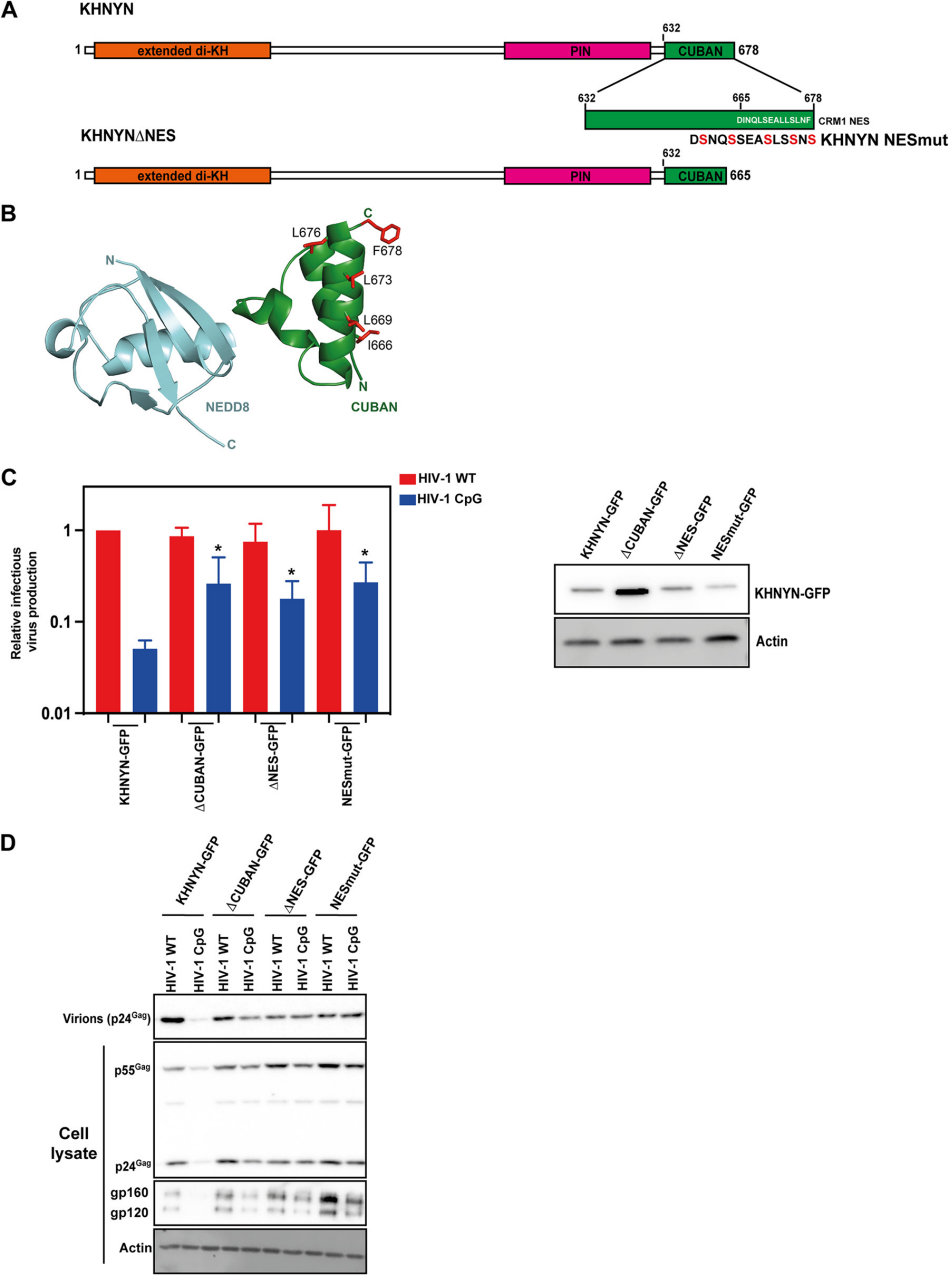
A nuclear export signal present in the CUBAN domain is required for KHNYN antiviral activity. (A) Schematic representation of the KHNYN CUBAN domain and nuclear export signal. The residues that were mutated in KHNYN-NESmut are shown in red. (B) Cartoon representation of the NEDD8-CUBAN complex structure (PDB: 2N7K). Residues in the NES that were mutated to serine are shown as sticks in red. (C) Left panel: Infectious virus production from KHNYN CRISPR HeLa cells stably expressing wild-type KHNYN-GFP, KHNYNΔCUBAN-GFP, KHNYNΔNES-GFP or KHNYN-NESmut-GFP transfected with HIV-1_WT_ or HIV-1_CpG_. Each bar shows the average value of three independent experiments normalized to the value obtained for wild-type KHNYN co-transfected with pHIV-1_WT_. *, *P* < 0.05 as determined by a two-tailed *unpaired t test* comparing each mutant KHNYN-GFP sample to wild-type KHNYN-GFP. Right panel: Representative Western blot showing KHNYN-GFP protein levels. (D) Representative Western blots for virion production in the media as well as Gag and Env in cell lysates.

There are several types of NESs in CRM1 cargo proteins with different spacing of hydrophobic residues that fit into 5 pockets in CRM1 including the Rev-type NES and the PKI-type NES (56). To identify potential NESs in KHNYN, we used the Wregex tool (61), which identified a putative NES at the C-terminus of the CUBAN domain. This NES has hydrophobic residues with the PKI-type NES spacing (residues 669 to 678, LSEALLSLNF, amino acids predicted to bind CRM1 are underlined). Of note, this tool only identified positions 1 to 4 for the predicted CRM1 NES and did not identify the more recently identified position 0 (56, 61). For a PKI-type NES, position 0 is 3 amino acids upstream of position 1 and is preferentially preceded by an acidic residue (56). The C-terminal NES in KHNYN fits this consensus perfectly, with the full NES predicted to be DINQLSEALLSLNF (Fig. 6A). Interestingly, L676 and F678 at positions 3 and 4 in the NES were among the top 5 residues with the largest differential in the ConSurf analysis comparing the mammal/reptile KHNYN MSA to the mammal/reptile/bony fish MSA (Fig. 2D), suggesting that they evolved in the tetrapod lineage. The NES is located in the third helix of the CUBAN domain (Fig. 6B), which does not contain any of the residues that interact with NEDD8.

**FIG 7:**
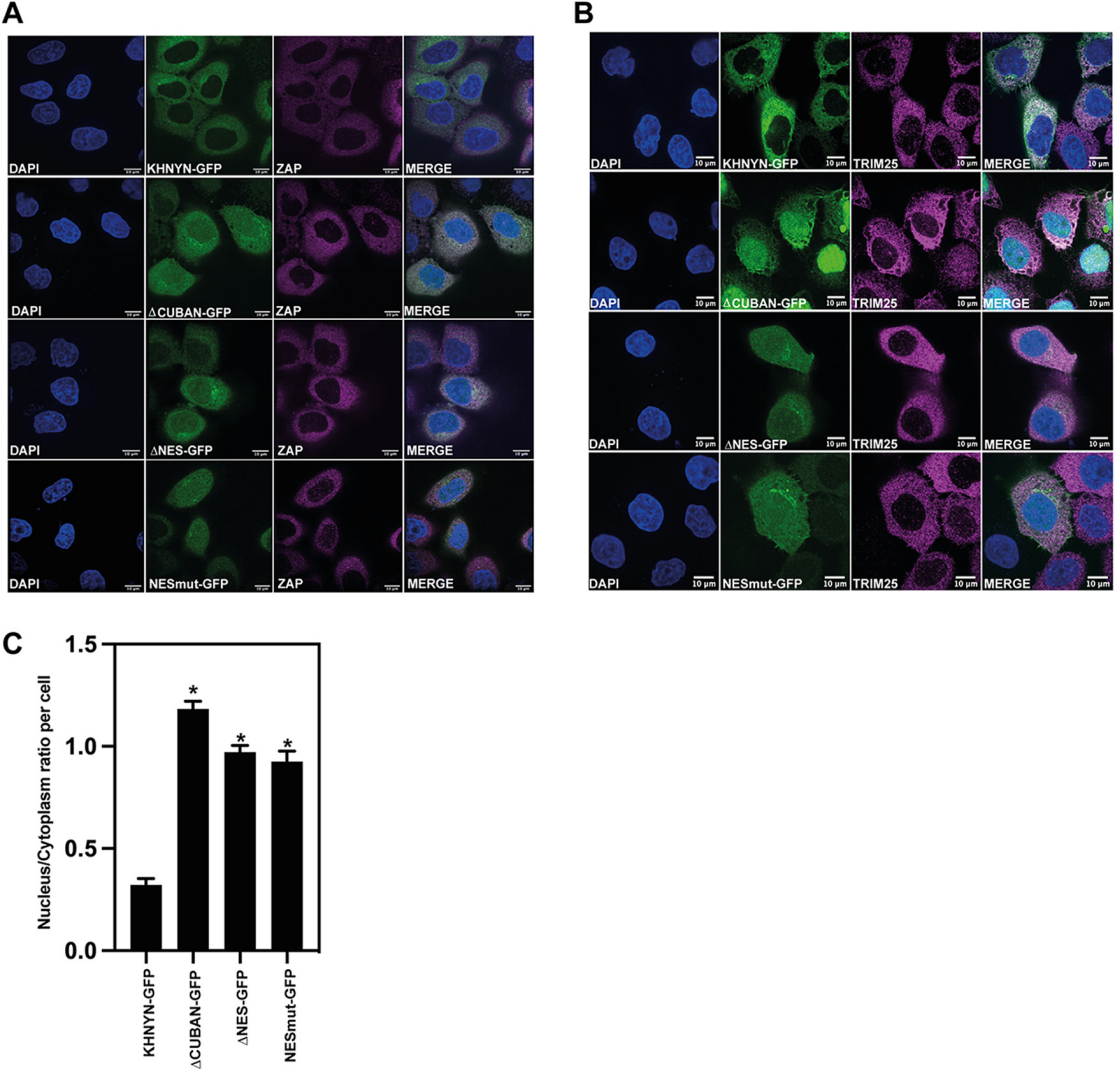
The nuclear export signal in the C-terminus of the CUBAN domain is required for KHNYN cytoplasmic location. (A to B) Confocal microscopy images of the KHNYN-GFP cell lines (green) co-stained for endogenous ZAP (magenta) (A) or TRIM25 (magenta) (B), scale bar is 10 μm. (C) Signal quantification per cell (50 cells total per condition) of the ratio of KHNYN nuclear to cytoplasmic distribution in the KHNYN-GFP cell lines. *, *P* < 0.05 as determined by a two-tailed *unpaired t test* comparing the nuclear/cytoplasmic ratio between each sample.

To test the functional role of the NES in KHNYN, we made stable cell lines expressing KHNYNΔNES-GFP and KHNYN-NESmut-GFP. KHNYN-NESmut-GFP has all 5 amino acids predicted to directly bind CRM1 mutated to serine and, in KHNYNΔNES-GFP, the NES sequence was deleted (Fig. 6A). Deleting or mutating the NES decreased KHNYN antiviral activity and increased its nuclear localization, similar to KHNYN-ΔCUBAN (Fig. 6C and D, and Fig. 7A to C). This suggests that the loss of antiviral activity for KHNYNΔCUBAN is due to the deletion of the C-terminal NES in the CUBAN domain. While deletion or mutation of the NES localizes KHNYN to the nucleus, this has no effect on ZAP or TRIM25 localization (Fig. 7A and B).

### Evolution of the KHNYN CUBAN domain.

**FIG 8:**
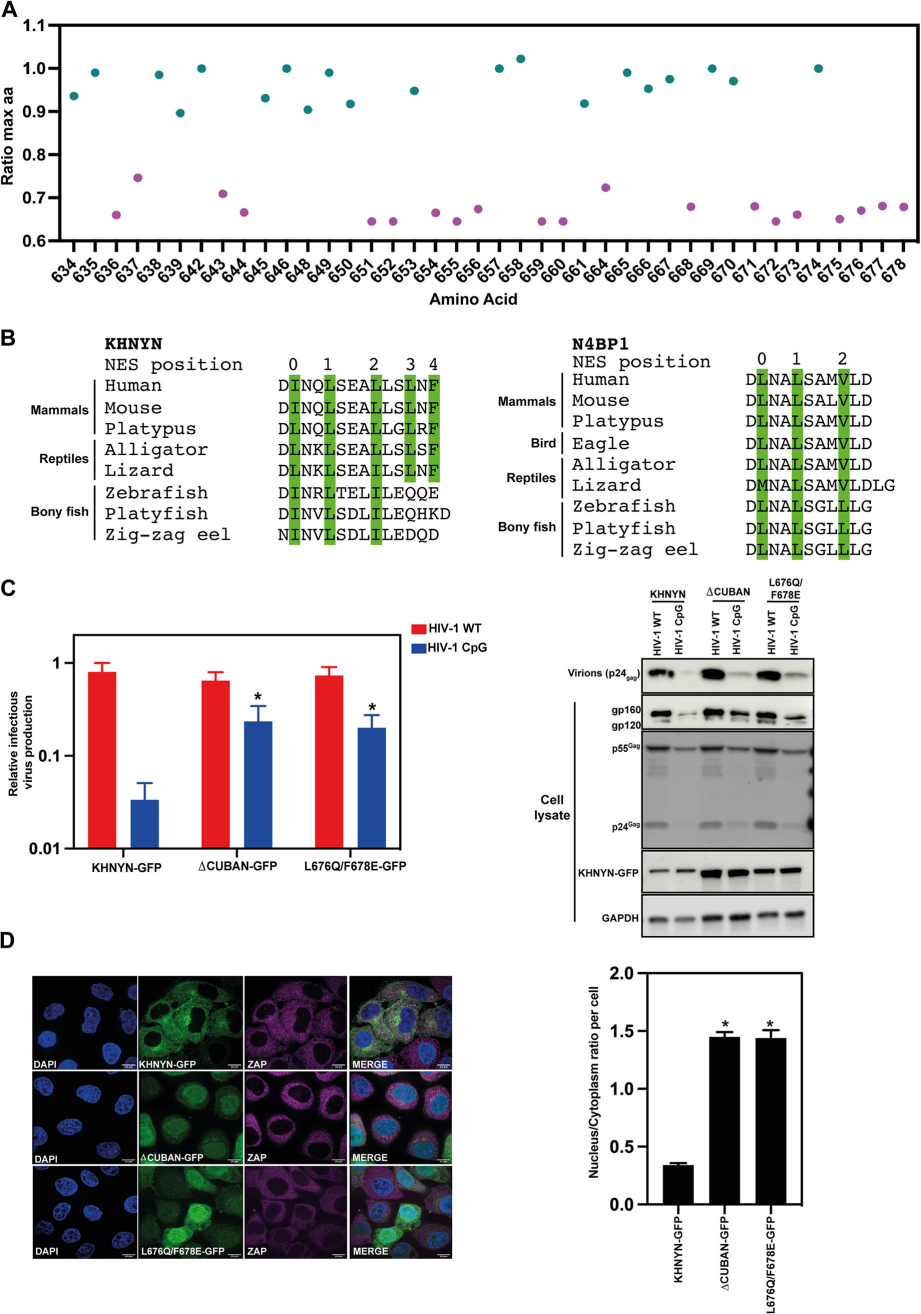
Positions 3 and 4 in the KHNYN NES in the CUBAN domain evolved at a similar time as ZAP in tetrapods and are required for KHNYN antiviral activity and cytoplasmic localization. (A) Ratio of the percentage of the maximum amino acid present at each position in the CUBAN domain in the mammal/reptile MSA to the mammal/reptile/bony fish MSA. Residues with a ratio > 0.85 are colored cyan and residues with a ratio < 0.75 are colored magenta. Residues that were not highly conserved in the mammal/reptile MSA (ConSurf categories 7, 8, and 9) are not shown. (B) Alignment of the C-terminal NES residues of KHNYN and N4BP1 from selected species. Residues in PKI-like NES positions 0 to 4 are highlighted in green. (C) Left panel: Infectious virus production from KHNYN CRISPR HeLa cells stably expressing wild-type KHNYN-GFP, KHNYNΔCUBAN-GFP or KHNYN L676Q/F678E transfected with HIV-1_WT_ or HIV-1_CpG_. Each bar shows the average value of three independent experiments normalized to the value obtained for wild-type KHNYN co-transfected with pHIV-1_WT_. *, *P* < 0.05 as determined by a two-tailed unpaired t test comparing each mutant KHNYN-GFP sample to wild-type KHNYN-GFP. Right panel: Representative Western blots for virion production in the media as well as Gag, Env and GFP in cell lysates. (D) Left panel: Confocal microscopy images of the KHNYN-GFP cell lines (green) co-stained for endogenous ZAP (magenta), scale bar is 10 μm. Right panel: Signal quantification per cell (50 cells total per condition) of the ratio of KHNYN nuclear to cytoplasmic distribution in the KHNYN-GFP cell lines. *, *P* < 0.05 as determined by a two-tailed unpaired t test comparing nuclear/cytoplasmic ratio between each sample.

To further analyze how the CUBAN domain in KHNYN has evolved since ZAP emerged, we calculated the ratio of the percentage of the dominant amino acid at a specific site in the mammal/reptile MSA to the percentage of the dominant residue at that site in the mammal/reptile/bony fish MSA (see materials and methods for more detail, Data set S1). We then plotted the ratio for the highly conserved residues in the in the mammal/reptile MSA (Fig. 8A, categories 7, 8, and 9 in the ConSurf analysis, Data set S1). Two clear distributions of the amino acids are present: residues that are highly conserved in KHNYN from bony fish to mammals (ratio > 0.85, colored in cyan) and residues that are conserved only in the mammal and reptile lineage (ratio < 0.75, colored in magenta) (Fig. 8A). Inspection of the CUBAN domain structure (38) shows that many of the highly conserved residues in mammals, reptiles and bony fishes are likely essential for the overall fold including T635, L638, R639, F646, G648, V653, L657, P661, N667, and L669. Many of these residues are also conserved in cartilaginous fishes, lampreys, lancelets as well as invertebrates (echinoderms and molluscs) (Fig. S3), highlighting that a CUBAN-like domain is an ancestral feature of the N4BP1-like proteins.

Several of the residues that are highly conserved in mammal and reptile KHNYN orthologs, but not those in bony fish, are part of the basic binding site in the CUBAN domain that interacts with NEDD8, including H651 and K652. In addition, several of the residues that make up the NES are conserved in mammals and reptiles but not in bony fish. Looking at the 5 residues in the NES that interact with CRM1, interesting patterns emerge. I666 and L669 at positions 0 and 1 in the NES are highly conserved in KHNYN orthologs and are also conserved as a bulky hydrophobic residue in N4BP1 and N4BP1-like proteins (Fig. 8B and Fig. S3). Isoleucine and leucine are the strongest amino acids for a PKI-type NES at positions 0 and 1, respectively (56). At position 2 in the NES, a bulky hydrophobic residue is also often present in KHNYN and N4BP1 orthologs (Fig. 8B and Fig. S3). However, it is typically a leucine in tetrapod KHNYN orthologs in contrast to bony fish KHNYN orthologs, N4BP1 or N4BP1-like proteins, where it is usually an isoleucine or valine, both of which are weaker in the context of a PKI-type NES (56). As noted above, L676 and F678 at positions 3 and 4 in the NES were among the residues that changed the most in the ConSurf analysis in Fig. 2D, indicating that they evolved in the KHNYN tetrapod lineage. L676 is not present in most bony fish KHNYN orthologs, N4BP1 orthologs and N4BP1-like proteins. (Fig. 8B and Fig. S3). F678 is not found in any of the sequences analyzed outside of tetrapod KHNYN orthologs. To determine whether the NES is functional without leucine and phenylalanine at positions 3 and 4, we mutated L676 and F678 to the residues found in zebrafish KHNYN (L676Q, F676E) and made stable cell lines expressing these proteins with a GFP tag. As expected, KHNYN L676Q/F678E-GFP had a substantial loss of antiviral activity and was localized in the nucleus (Fig. 8C and D). This indicates that the tetrapod-specific changes in the C-terminus of KHNYN created a NES that allows it to act as a ZAP cofactor.

### The nuclear export signal in the CUBAN domain is required for KHNYN to interact with ZAP-L.

**FIG 9:**
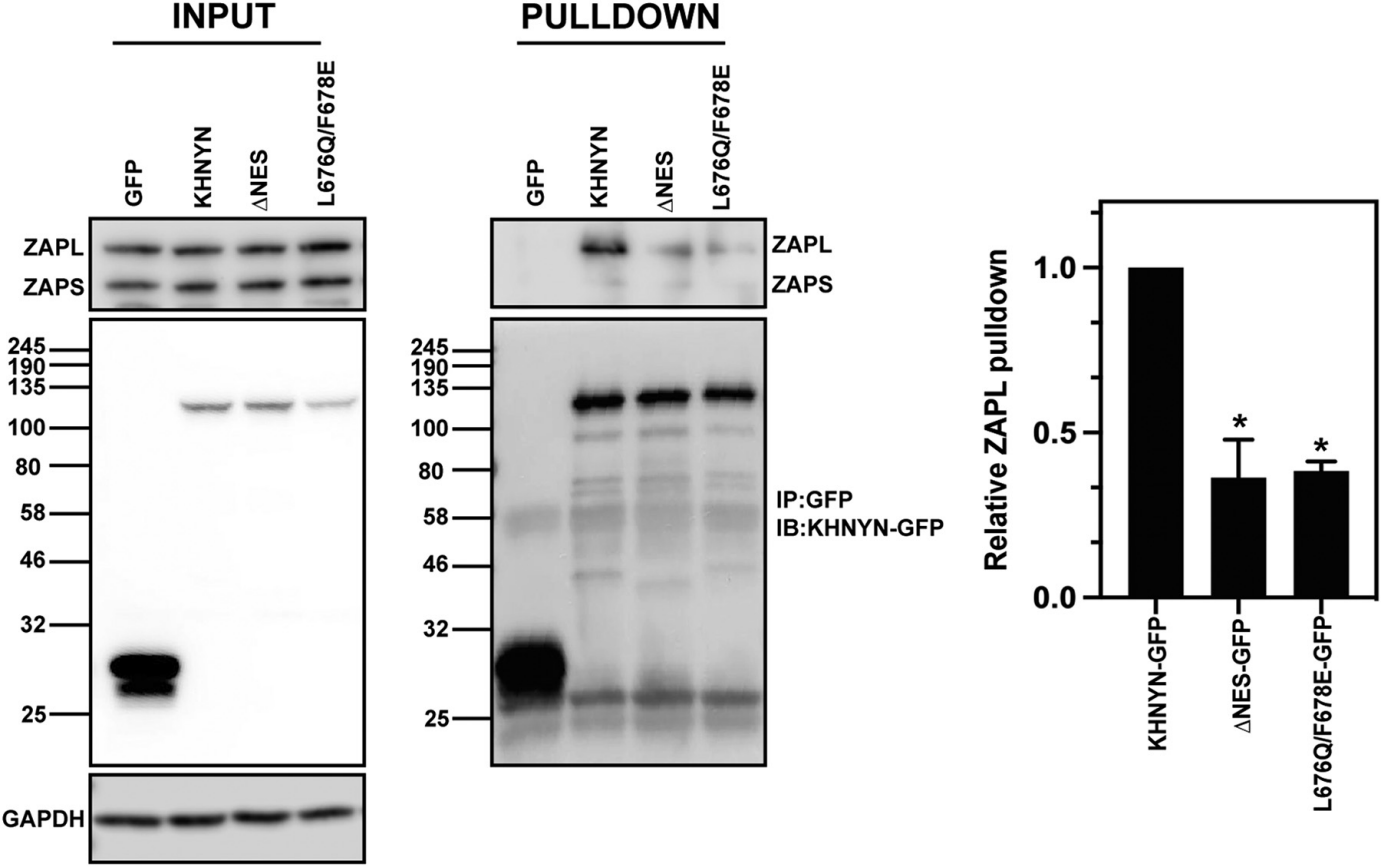
The NES in the KHNYN CUBAN domain promotes its interaction with ZAP. Left panel: GFP control, KHNYN-GFP and KHNYNΔNES-GFP HeLa cells were lysed and immunoblotted for GFP and endogenous ZAP. Middle panel: GFP was immunoprecipitated in each cell lysate and blotted for GFP or endogenous ZAP. Right panel: The amount of ZAP-L immunoprecipitated relative to the KHNYN-GFP sample is presented in a bar graph, N = 3. *, *P* = <0.05 as determined by a two-tailed unpaired t test.

There are 2 predominant isoforms for ZAP, ZAP-L and ZAP-S (11, 62). ZAP-L contains a C-terminal S-farnesylation modification that localizes it to the cytoplasmic endomembrane system while ZAP-S appears to be diffuse in the cytosol (44, 63–65). ZAP-L has greater antiviral activity than ZAP-S against some viruses, including HIV-1_CpG_, and this requires the S-farnesylation post-translational modification (44, 62–64). ZAP-L interacts with KHNYN more efficiently than ZAP-S and the interaction between ZAP-L and KHNYN is decreased when the CaaX box in ZAP-L that mediates S-farnesylation is mutated (44). Therefore, the NES in KHNYN may be necessary for its antiviral activity because it is required for KHNYN to traffic to the cytoplasm so it can interact with ZAP-L. To determine if the nuclear export signal in KHNYN is required for it to interact with ZAP, we performed co-immunoprecipitation experiments in the KHNYN-GFP, KHNYNΔNES-GFP, and KHNYN L676Q/F678E-GFP cell lines, though it should be noted that the interaction between KHNYN and ZAP could occur post-lysis. Immunoprecipitation of KHNYN-GFP pulled down ZAP-L but little ZAP-S (Fig. 9). Importantly, KHNYNΔNES-GFP and KHNYN L676Q/F678E-GFP immunoprecipitated less ZAP-L than wild-type KHNYN (Fig. 9). This suggests that when KHNYN is localized in the nucleus, it cannot interact with ZAP-L at the cytoplasmic endomembrane system and therefore its nuclear export signal is required for this interaction and its activity as a ZAP cofactor.

## DISCUSSION

The development of the interferon system has led to extensive evolution in many genes (66). Many ISGs have been shown to have undergone periods of rapid evolution and a panel of ISGs have been shown to evolve faster than interferon-induction genes or a panel of random genes (67). However, little is known about the evolution of non-ISGs that encode ISG cofactors and how these genes evolve after the emergence of an ISG. Importantly, this evolutionary signature is likely to be different from the signature induced by a viral countermeasure to an ISG. Instead of a positive natural selection signature, evolutionary changes that allow a protein to act as an ISG cofactor are likely to be fixed after they develop or to co-evolve with the ISG.

KHNYN appears to have undergone extensive evolution after ZAP emerged in tetrapods. While there could be several different evolutionary forces that led to this, changes that allow it to act as a ZAP cofactor could be responsible for some of the differences in specific residues between the bony fish and tetrapod lineages. In this study, we focused on how the CUBAN domain evolved and identified a CRM1-dependent NES present in mammalian and reptile KHNYN orthologs that is required for its nuclear-cytoplasmic shuttling, interaction with ZAP and antiviral activity. The decrease in the interaction between KHNYN and ZAP when the NES is mutated or deleted is likely at least partially due to it being sequestered in the nucleus, which leads to low levels of cytoplasmic KHNYN. However, it is possible that the nuclear-cytoplasmic trafficking pathway also makes KHNYN more competent to act as a ZAP cofactor, such as by altering its post-translational modifications or its interaction partners. The NES has evolved in the tetrapod KHNYN lineage, with the most substantial changes in positions 3 and 4. When these positions were mutated to the residues found in zebrafish KHNYN, the protein was localized in the nucleus, did not efficiently interact with ZAP-L and had little antiviral activity. Once the full NES evolved, it became fixed in the reptile and mammal lineages.

The low abundance of KHNYN appears to be at least partly due to its CUBAN domain, which mediates an interaction with neddylated cullin-RING E3 ubiquitin ligases (38). Mutation of residues in this domain that mediate binding to NEDD8 led to a substantial increase in KHNYN abundance, though this does not affect its antiviral activity. KHNYN protein levels could be tightly regulated to prevent off-target endoribonuclease activity, which would be detrimental to cellular gene expression, thus necessitating turnover by cullin-RING E3 ubiquitin ligases. In addition, KHNYN could have additional cellular functions beyond acting as a ZAP cofactor since KHNYN orthologs are present in bony fishes, which do not have known ZAP orthologs. It should be noted that the function of N4BP1 and KHNYN in fishes is not known.

ZAP subcellular localization appears to regulate its antiviral activity against several viruses in that ZAP-L, which contains a S-farnesylation motif that targets it to the endomembrane system, mediates more potent restriction than ZAP-S (44, 62–64). This correlates with preferential KHNYN and TRIM25 binding to ZAP-L compared to ZAP-S, even though the binding sites for KHNYN and TRIM25 are present in both isoforms (8, 20, 22, 44). KHNYN subcellular localization also appears to be important in that its CRM1 NES is required for antiviral activity. How KHNYN is targeted to the nucleus is not clear and we have not identified a canonical nuclear localization signal (NLS) in it. However, KHNYN could be trafficked into the nucleus by interacting with other proteins that contain an NLS. The timing for how KHNYN interacts with ZAP relative to ZAP binding to target RNA is not known. One possibility is that cytoplasmic ZAP-L molecules bind KHNYN prior to binding RNA, leading to a pre-formed antiviral complex. However, KHNYN appears to be limiting for ZAP antiviral activity because it is expressed at low levels and its overexpression potently promotes restriction of CpG-enriched HIV-1 (22, 48, 50, 57). Thus, only a small pool of ZAP molecules may be bound to KHNYN under steady-state conditions. Another possibility is that KHNYN cycles through the nucleus and cytoplasm and only interacts with ZAP after it binds RNA. This could act as a regulatory mechanism to allow endonucleolytic cleavage only for RNAs that have ZAP bound to them with a particular stoichiometry or structure. Therefore, in addition to its low abundance, nuclear localization of KHNYN could regulate its activity by preventing it from interacting with ZAP-bound RNAs that are not bona fide targets.

## MATERIALS AND METHODS

### Plasmids and cell lines.

HeLa, HEK293T, and TZM-bl cells were maintained in high glucose DMEM supplemented with GlutaMAX (Thermo Fisher Scientific), 10% fetal bovine serum, 100 U/mL penicillin and 100 mg/mL streptomycin, and incubated with 5% CO_2_ at 37°C. Control CRISPR and KHNYN CRISPR HeLa cells were previously described (22). HIV-1_NL4-3_ (pHIV-1_WT_) and HIV*env*_86-561_CpG (pHIV-1_CpG_) in pGL4 were previously described (22, 68). The CRISPR-resistant pKHNYN-FLAG plasmid has been previously described (22), and specific mutations were cloned into it using site-specific mutagenesis. CRISPR-resistant KHNYN-GFP constructs were made using a flexible “GGGGSGGGGSGGGG” linker between KHNYN and GFP in the context of the murine leukemia virus (MLV)-based retroviral vector MIGR1 with the GFP replaced by the Blasticidin S-resistance gene (69). Mutant KHNYN-GFP constructs were made by either synthesizing the sequence or introducing point mutations using PCR. All primers and synthesized DNA sequences were purchased from Eurofins Genomics and all PCRs were performed using Q5 High-Fidelity (New England Biolabs). Stable CRISPR KHNYN HeLa cells expressing CRISPR-resistant KHNYN-GFP, KHNYNmutNEDD8-GFP, KHNYNΔCUBAN-GFP, KHNYNΔNES-GFP, KHNYN-NESmut-GFP and KHNYN L676Q/L678E were produced by retroviral vector transduction.

### Transfections and infections.

HeLa cells were seeded in 24-well plates at 70% confluence. Cells were transfected according to the manufacturer’s instructions using TransIT-LT1 (Mirus) at the ratio of 3 μL TransIT- LT1 to 1 μg DNA. For the HIV experiments, 0.5 μg pHIV_WT_ or pHIV_CpG_ and the designated amount of KHNYN-FLAG or GFP-FLAG for a total of 1 μg DNA were transfected. After 24 h posttransfection, the culture media was replaced with fresh media. For HIV-1_WT_ or HIV-1_CpG_ infection of HeLa cells, viral stocks were produced by co-transfecting pHIV-1_WT_ or pHIV-1_CpG_ with pVSV-G (70) into HEK293T ZAP CRISPR cells (48), and titrated on TZM-bl cells.

### Analysis of protein expression by immunoblotting.

After 48 h posttransfection, the HeLa cells were lysed in Laemmli buffer and heated at 95°C for 10 min. The culture supernatant was filtered through a 0.45 μm filter and virions were pelleted by centrifugation for 2 h at 20,000 × *g* through a 20% sucrose cushion in phosphate-buffered saline (PBS). Viral pellets were resuspended in 2X Laemmli buffer. Cell lysates and virion lysates were resolved on 8% to 16% Mini-Protean TGX precast gels (Bio-Rad), transferred onto nitrocellulose membranes (GE Healthcare), and blocked in 5% nonfat milk in PBS with 0.1% Tween 20. Primary antibodies were incubated overnight at 4°C followed by 3 washes in PBS with 0.1% Tween 20 and the corresponding secondary antibody was incubated for 1 h. Proteins were visualized by LI-COR (Odyssey Fc) measuring secondary antibody fluorescence or using Amersham ECL Prime Western Blotting Detection reagent (GE Lifesciences) for HRP-linked antibodies with an ImageQuant (LAS8000 Mini). Primary and secondary antibodies used in this study: 1:50 HIV anti-p24Gag (71) (Mouse), 1:3000 anti-HIV gp160/120 (Rabbit, ADP421; Centralized Facility for AIDS Reagents [CFAR]), 1:5000 anti-HSP90 (Rabbit, GeneTex, GTX109753), 1:1000 anti-FLAG (DYKDDDDK, Rabbit, Cell Signaling, 14793), 1:2000 anti-beta Actin (Mouse, Abcam; Ab6276), 1:5000 anti-ZAP (Rabbit, Abcam, ab154680), 1:1000 anti-GFP (Mouse. Roche 11814460001), 1:5000 anti-rabbit HRP (Cell Signaling Technology, 7074), 1:5000 anti-mouse HRP (Cell Signaling Technology, 7076), 1:5000 anti-mouse IRDye 680RD (LI-COR, 926–68070), and 1:5000 anti-rabbit IRDye 800CW (LI-COR, 926–32211).

### TZM-bl infectivity assay.

The TZM-bl indicator cell line was used to quantify the amount of infectious virus (72–74). Briefly, cells were seeded in 24-well plates and infected by incubation with virus stocks. After 48 h postinfection, the cells were lysed and infectivity was measured by β-galactosidase expression using the Galacto-Star System following manufacturer’s instructions (Applied Biosystems). β-galactosidase activity was quantified as relative light units per second using a PerkinElmer Luminometer.

### Immunoprecipitation assays.

HeLa cells stably expressing wild-type KHNYN-GFP wild-type or mutant versions were seeded in 6-well plates for 24 h prior to immunoprecipitation. The cells were lysed on ice in lysis buffer (0.5% NP-40, 150 mM KCl, 10 mM HEPES pH 7.5, 3 mM MgCl_2_) supplemented with complete Protease inhibitor cocktail tablets (Sigma-Aldrich). The lysates were incubated on ice for 1 h and centrifuged at 20,000 × *g* for 15 min at 4°C. A total of 50 μL of the post-nuclear supernatant was saved as the input lysate and 450 μL was incubated with 5 μg of anti-GFP antibody (Roche 11814460001) for 1 h at 4°C. Protein G Dynabeads (Invitrogen) were then added and incubated overnight at 4°C with rotation. The lysates were washed four times with wash buffer (0.05% NP-40, 150 mM KCl, 10 mM HEPES pH 7.5, 3 mM MgCl_2_) before the bound proteins were eluted with 2X Laemmli buffer and boiled for 10 min. Protein expression was analyzed by Western blotting as described above.

### Microscopy.

HeLa cells stably expressing wild-type KHNYN-GFP or versions with specific mutations were seeded in pretreated 24-well plates 24 h prior to immunostaining. The cells were fixed with 4% paraformaldehyde for 20 min at room temperature, washed once with 1X PBS, washed once in 10 mM glycine and then permeabilized for 15 min in 1% BSA and 0.1% Triton-X in PBS. Rabbit anti-ZAP (1:500) or rabbit anti-TRIM25 (1:500) antibodies were diluted in 1X PBS/0.01% Triton-X and the cells were stained for 1 h at room temperature. The cells were then washed three times in PBS/0.01% Triton-X and incubated with Alexa Fluor 594 anti-rabbit (Molecular Probes, 1:500 in 1X PBS/0.01% Triton-X) for 45 min in the dark. Finally, the coverslips were washed three times with 1X PBS/0.01% Triton X-100 and then mounted on slides using Prolong Diamond Antifade Mountant with DAPI (Invitrogen). For the Leptomycin B treatment experiments, HeLa cells stably expressing KHNYN-GFP wild-type or mutants were seeded in pretreated 24-well plates 24 h prior to a 4 h treatment with 50 nM Leptomycin B or DMSO at 37°C. After treatment, the cells were fixed and immunostained as described above. Imaging was performed on a Nikon Eclipse Ti Inverted Microscope, equipped with a Yokogawa CSU/X1-spinning disk unit, under 100x objective and laser wavelengths of 405 nm and 564 nm. Image processing and co-localization analyses were performed with Image J (Fiji) software.

### Phylogenetic analysis of KHNYN and N4BP1, AlphaFold models, and NES prediction.

Amino acid sequences for KHNYN and N4BP1 were obtained from NCBI Gene, checked manually to ensure they were full-length sequences and aligned using ClustalOmega (75). The resulting alignment file was used to generate a maximum likelihood phylogenetic tree with RAxMLin the DIVEIN web server (76) using the LG substitution model and 100-bootstrap test. N4BP1-like sequences from the Californian sea hare (*A. californica*), and the Crown-of-thorns Starfish (*A. planci*) were used as outgroups to root the tree. The resulting tree was visually presented and annotated using the interactive Tree of life (iTol) (77).

KHNYN protein sequences for the ConSurf analysis (39, 40, 78) were obtained from NCBI Gene, checked manually to ensure they were full-length sequences and aligned with MUSCLE (79) to produce multiple sequence alignments (MSAs). The placental mammal MSAs contained 109 sequences, the bony fish MSA contained 72 sequences, the mammal/reptile MSA contained 131 sequences and the mammal/reptile/bony fish MSA contained 203 sequences. These bespoke MSAs were used with the ConSurf Server at https://consurf.tau.ac.il/. Either human or zebrafish KHNYN was used as the Query Sequence. The settings used were the Bayesian calculation method and ‘Best model’ evolutionary substitution model. ConSurf provided several different outputs that were then used for the analysis and specific details for these outputs can be found at https://consurf.tau.ac.il/overview.php. First, the conservation score represents the evolutionary conservation at each residue, with the lowest score indicating the most conserved position in the sequence in the context of the MSA. The scores are normalized with the average score being 0 and a standard deviation of 1. Second, the conservation scores above and below average are each divided into 4.5 equal intervals to produce the 9 categories with the most conserved residues in category 9 and the least conserved in category 1. Third, the dominant amino acid at each position in the MSA is identified as well as the percentage of sequences with this amino acid. We determined the ratio of the percentage of the dominant amino acid at each position by dividing the percentage in the mammal/reptile MSA by the percentage in the mammal/reptile/bony fish MSA. A residue that is 100% conserved in each MSA will have a ratio of 1. Only residues in the CUBAN domain that were highly conserved in the mammal/reptile MSA (ConSurf categories 7, 8, and 9) were analyzed further.

The AlphaFold models for N4BP1 and KHNYN are available in the AlphaFold Protein Structure Database (36, 37) at https://alphafold.ebi.ac.uk/. We used the following models: human KHNYN (https://alphafold.ebi.ac.uk/entry/O15037), mouse KHNYN (https://alphafold.ebi.ac.uk/entry/Q80U38), zebrafish KHNYN (https://alphafold.ebi.ac.uk/entry/F1QVW5), human N4BP1 (https://alphafold.ebi.ac.uk/entry/O75113), mouse N4BP1 (https://alphafold.ebi.ac.uk/entry/Q6A037), and zebrafish N4BP1 (https://alphafold.ebi.ac.uk/entry/Q1LVK9). The NES was identified using the Wregex tool with the NES/CRM1 motif and the relaxed configuration (61).

### Statistical analysis.

Statistical significance was determined using unpaired two-tailed t tests in GraphPad. Data are represented as mean ± standard deviation and significance was ascribed to *P* values < 0.05.

## References

[1] tenOever BR. 2016. The evolution of antiviral defense systems. Cell Host Microbe 19:142–149. 10.1016/j.chom.2016.01.006. 26867173

[2] Shabalina SA, Koonin EV. 2008. Origins and evolution of eukaryotic RNA interference. Trends Ecol Evol 23:578–587. 10.1016/j.tree.2008.06.005. 18715673 PMC2695246

[3] Maillard PV, Veen AG, Poirier EZ, Reis e Sousa C. 2019. Slicing and dicing viruses: antiviral RNA interference in mammals. EMBO J 38:e100941. 10.15252/embj.2018100941. 30872283 PMC6463209

[4] Ablasser A, Hur S. 2020. Regulation of cGAS- and RLR-mediated immunity to nucleic acids. Nat Immunol 21:17–29. 10.1038/s41590-019-0556-1. 31819255

[5] Lazear HM, Schoggins JW, Diamond MS. 2019. Shared and distinct functions of type I and type III interferons. Immunity 50:907–923. 10.1016/j.immuni.2019.03.025. 30995506 PMC6839410

[6] Secombes CJ, Zou J. 2017. Evolution of interferons and interferon receptors. Front Immunol 8:209. 10.3389/fimmu.2017.00209. 28303139 PMC5332411

[7] Schoggins JW. 2019. Interferon-stimulated genes: what do they all do? Annu Rev Virol 6:567–584. 10.1146/annurev-virology-092818-015756. 31283436

[8] Goncalves-Carneiro D, Takata MA, Ong H, Shilton A, Bieniasz PD. 2021. Origin and evolution of the zinc finger antiviral protein. PLoS Pathog 17:e1009545. 10.1371/journal.ppat.1009545. 33901262 PMC8102003

[9] Shaw AE, Hughes J, Gu Q, Behdenna A, Singer JB, Dennis T, Orton RJ, Varela M, Gifford RJ, Wilson SJ, Palmarini M. 2017. Fundamental properties of the mammalian innate immune system revealed by multispecies comparison of type I interferon responses. PLoS Biol 15:e2004086. 10.1371/journal.pbio.2004086. 29253856 PMC5747502

[10] Levraud JP, Jouneau L, Briolat V, Laghi V, Boudinot P. 2019. IFN-stimulated genes in zebrafish and humans define an ancient arsenal of antiviral immunity. J Immunol 203:3361–3373. 10.4049/jimmunol.1900804. 31732531

[11] Gao G, Guo X, Goff SP. 2002. Inhibition of retroviral RNA production by ZAP, a CCCH-type zinc finger protein. Science 297:1703–1706. 10.1126/science.1074276. 12215647

[12] Bick MJ, Carroll JW, Gao G, Goff SP, Rice CM, MacDonald MR. 2003. Expression of the zinc-finger antiviral protein inhibits alphavirus replication. J Virol 77:11555–11562. 10.1128/jvi.77.21.11555-11562.2003. 14557641 PMC229374

[13] Guo X, Carroll JW, Macdonald MR, Goff SP, Gao G. 2004. The zinc finger antiviral protein directly binds to specific viral mRNAs through the CCCH zinc finger motifs. J Virol 78:12781–12787. 10.1128/JVI.78.23.12781-12787.2004. 15542630 PMC525010

[14] Zhu Y, Wang X, Goff SP, Gao G. 2012. Translational repression precedes and is required for ZAP-mediated mRNA decay. EMBO J 31:4236–4246. 10.1038/emboj.2012.271. 23023399 PMC3492732

[15] Takata MA, Goncalves-Carneiro D, Zang TM, Soll SJ, York A, Blanco-Melo D, Bieniasz PD. 2017. CG dinucleotide suppression enables antiviral defence targeting non-self RNA. Nature 550:124–127. 10.1038/nature24039. 28953888 PMC6592701

[16] Ficarelli M, Neil SJD, Swanson CM. 2021. Targeted restriction of viral gene expression and replication by the ZAP antiviral system. Annu Rev Virol 8:265–283. 10.1146/annurev-virology-091919-104213. 34129371

[17] Guo X, Ma J, Sun J, Gao G. 2007. The zinc-finger antiviral protein recruits the RNA processing exosome to degrade the target mRNA. Proc Natl Acad Sci USA 104:151–156. 10.1073/pnas.0607063104. 17185417 PMC1765426

[18] Chen G, Guo X, Lv F, Xu Y, Gao G. 2008. p72 DEAD box RNA helicase is required for optimal function of the zinc-finger antiviral protein. Proc Natl Acad Sci USA 105:4352–4357. 10.1073/pnas.0712276105. 18334637 PMC2393818

[19] Zhu Y, Chen G, Lv F, Wang X, Ji X, Xu Y, Sun J, Wu L, Zheng YT, Gao G. 2011. Zinc-finger antiviral protein inhibits HIV-1 infection by selectively targeting multiply spliced viral mRNAs for degradation. Proc Natl Acad Sci USA 108:15834–15839. 10.1073/pnas.1101676108. 21876179 PMC3179061

[20] Li MM, Lau Z, Cheung P, Aguilar EG, Schneider WM, Bozzacco L, Molina H, Buehler E, Takaoka A, Rice CM, Felsenfeld DP, MacDonald MR. 2017. TRIM25 enhances the antiviral action of zinc-finger antiviral protein (ZAP). PLoS Pathog 13:e1006145. 10.1371/journal.ppat.1006145. 28060952 PMC5245905

[21] Zheng X, Wang X, Tu F, Wang Q, Fan Z, Gao G. 2017. TRIM25 is required for the antiviral activity of zinc finger antiviral protein. J Virol 91:e00088-17. 10.1128/JVI.00088-17. 28202764 PMC5391446

[22] Ficarelli M, Wilson H, Pedro Galao R, Mazzon M, Antzin-Anduetza I, Marsh M, Neil SJ, Swanson CM. 2019. KHNYN is essential for the zinc finger antiviral protein (ZAP) to restrict HIV-1 containing clustered CpG dinucleotides. Elife 8:e46767. 10.7554/eLife.46767. 31284899 PMC6615859

[23] Marco A, Marin I. 2009. CGIN1: a retroviral contribution to mammalian genomes. Mol Biol Evol 26:2167–2170. 10.1093/molbev/msp127. 19561090

[24] Plianchaisuk A, Kusama K, Kato K, Sriswasdi S, Tamura K, Iwasaki W. 2022. Origination of LTR retroelement-derived NYNRIN coincides with therian placental emergence. Mol Biol Evol 39. 10.1093/molbev/msac176. PMC9447858 35959649

[25] Nepravishta R, Ferrentino F, Mandaliti W, Mattioni A, Weber J, Polo S, Castagnoli L, Cesareni G, Paci M, Santonico E. 2019. CoCUN, a novel ubiquitin binding domain identified in N4BP1. Biomolecules 9:284. 10.3390/biom9070284. 31319543 PMC6681339

[26] Murillas R, Simms KS, Hatakeyama S, Weissman AM, Kuehn MR. 2002. Identification of developmentally expressed proteins that functionally interact with Nedd4 ubiquitin ligase. J Biol Chem 277:2897–2907. 10.1074/jbc.M110047200. 11717310

[27] Anantharaman V, Aravind L. 2006. The NYN domains: novel predicted RNAses with a PIN domain-like fold. RNA Biol 3:18–27. 10.4161/rna.3.1.2548. 17114934

[28] Sharma P, Murillas R, Zhang H, Kuehn MR. 2010. N4BP1 is a newly identified nucleolar protein that undergoes SUMO-regulated polyubiquitylation and proteasomal turnover at promyelocytic leukemia nuclear bodies. J Cell Sci 123:1227–1234. 10.1242/jcs.060160. 20233849 PMC2848111

[29] Oberst A, Malatesta M, Aqeilan RI, Rossi M, Salomoni P, Murillas R, Sharma P, Kuehn MR, Oren M, Croce CM, Bernassola F, Melino G. 2007. The Nedd4-binding partner 1 (N4BP1) protein is an inhibitor of the E3 ligase Itch. Proc Natl Acad Sci USA 104:11280–11285. 10.1073/pnas.0701773104. 17592138 PMC2040890

[30] Yamasoba D, Sato K, Ichinose T, Imamura T, Koepke L, Joas S, Reith E, Hotter D, Misawa N, Akaki K, Uehata T, Mino T, Miyamoto S, Noda T, Yamashita A, Standley DM, Kirchhoff F, Sauter D, Koyanagi Y, Takeuchi O. 2019. N4BP1 restricts HIV-1 and its inactivation by MALT1 promotes viral reactivation. Nat Microbiol 4:1532–1544. 10.1038/s41564-019-0460-3. 31133753

[31] Gitlin AD, Heger K, Schubert AF, Reja R, Yan D, Pham VC, Suto E, Zhang J, Kwon YC, Freund EC, Kang J, Pham A, Caothien R, Bacarro N, Hinkle T, Xu M, McKenzie BS, Haley B, Lee WP, Lill JR, Roose-Girma M, Dohse M, Webster JD, Newton K, Dixit VM. 2020. Integration of innate immune signalling by caspase-8 cleavage of N4BP1. Nature 587:275–280. 10.1038/s41586-020-2796-5. 32971525

[32] Shi H, Sun L, Wang Y, Liu A, Zhan X, Li X, Tang M, Anderton P, Hildebrand S, Quan J, Ludwig S, Moresco EMY, Beutler B. 2021. N4BP1 negatively regulates NF-kappaB by binding and inhibiting NEMO oligomerization. Nat Commun 12:1379. 10.1038/s41467-021-21711-5. 33654074 PMC7925594

[33] Li S, Wang L, Berman M, Kong YY, Dorf ME. 2011. Mapping a dynamic innate immunity protein interaction network regulating type I interferon production. Immunity 35:426–440. 10.1016/j.immuni.2011.06.014. 21903422 PMC3253658

[34] OhAinle M, Helms L, Vermeire J, Roesch F, Humes D, Basom R, Delrow JJ, Overbaugh J, Emerman M. 2018. A virus-packageable CRISPR screen identifies host factors mediating interferon inhibition of HIV. Elife 7:e39823. 10.7554/eLife.39823. 30520725 PMC6286125

[35] Matelska D, Steczkiewicz K, Ginalski K. 2017. Comprehensive classification of the PIN domain-like superfamily. Nucleic Acids Res 45:6995–7020. 10.1093/nar/gkx494. 28575517 PMC5499597

[36] Jumper J, Evans R, Pritzel A, Green T, Figurnov M, Ronneberger O, Tunyasuvunakool K, Bates R, Zidek A, Potapenko A, Bridgland A, Meyer C, Kohl SAA, Ballard AJ, Cowie A, Romera-Paredes B, Nikolov S, Jain R, Adler J, Back T, Petersen S, Reiman D, Clancy E, Zielinski M, Steinegger M, Pacholska M, Berghammer T, Bodenstein S, Silver D, Vinyals O, Senior AW, Kavukcuoglu K, Kohli P, Hassabis D. 2021. Highly accurate protein structure prediction with AlphaFold. Nature 596:583–589. 10.1038/s41586-021-03819-2. 34265844 PMC8371605

[37] Varadi M, Anyango S, Deshpande M, Nair S, Natassia C, Yordanova G, Yuan D, Stroe O, Wood G, Laydon A, Zidek A, Green T, Tunyasuvunakool K, Petersen S, Jumper J, Clancy E, Green R, Vora A, Lutfi M, Figurnov M, Cowie A, Hobbs N, Kohli P, Kleywegt G, Birney E, Hassabis D, Velankar S. 2022. AlphaFold protein structure database: massively expanding the structural coverage of protein-sequence space with high-accuracy models. Nucleic Acids Res 50:D439–D444. 10.1093/nar/gkab1061. 34791371 PMC8728224

[38] Castagnoli L, Mandaliti W, Nepravishta R, Valentini E, Mattioni A, Procopio R, Iannuccelli M, Polo S, Paci M, Cesareni G, Santonico E. 2019. Selectivity of the CUBAN domain in the recognition of ubiquitin and NEDD8. FEBS J 286:653–677. 10.1111/febs.14752. 30659753

[39] Ashkenazy H, Abadi S, Martz E, Chay O, Mayrose I, Pupko T, Ben-Tal N. 2016. ConSurf 2016: an improved methodology to estimate and visualize evolutionary conservation in macromolecules. Nucleic Acids Res 44:W344–350. 10.1093/nar/gkw408. 27166375 PMC4987940

[40] Berezin C, Glaser F, Rosenberg J, Paz I, Pupko T, Fariselli P, Casadio R, Ben-Tal N. 2004. ConSeq: the identification of functionally and structurally important residues in protein sequences. Bioinformatics 20:1322–1324. 10.1093/bioinformatics/bth070. 14871869

[41] Bi X, Wang K, Yang L, Pan H, Jiang H, Wei Q, Fang M, Yu H, Zhu C, Cai Y, He Y, Gan X, Zeng H, Yu D, Zhu Y, Jiang H, Qiu Q, Yang H, Zhang YE, Wang W, Zhu M, He S, Zhang G. 2021. Tracing the genetic footprints of vertebrate landing in non-teleost ray-finned fishes. Cell 184:1377–1391. 10.1016/j.cell.2021.01.046. 33545088

[42] Schoch CL, Ciufo S, Domrachev M, Hotton CL, Kannan S, Khovanskaya R, Leipe D, Mcveigh R, O’Neill K, Robbertse B, Sharma S, Soussov V, Sullivan JP, Sun L, Turner S, Karsch-Mizrachi I. 2020. NCBI Taxonomy: a comprehensive update on curation, resources and tools. Database (Oxford) 2020. 10.1093/database/baaa062. PMC7408187 32761142

[43] Meagher JL, Takata M, Goncalves-Carneiro D, Keane SC, Rebendenne A, Ong H, Orr VK, MacDonald MR, Stuckey JA, Bieniasz PD, Smith JL. 2019. Structure of the zinc-finger antiviral protein in complex with RNA reveals a mechanism for selective targeting of CG-rich viral sequences. Proc Natl Acad Sci USA 116:24303–24309. 10.1073/pnas.1913232116. 31719195 PMC6883784

[44] Kmiec D, Lista MJ, Ficarelli M, Swanson CM, Neil SJD. 2021. S-farnesylation is essential for antiviral activity of the long ZAP isoform against RNA viruses with diverse replication strategies. PLoS Pathog 17:e1009726. 10.1371/journal.ppat.1009726. 34695163 PMC8568172

[45] Xue G, Braczyk K, Goncalves-Carneiro D, Dawidziak DM, Sanchez K, Ong H, Wan Y, Zadrozny KK, Ganser-Pornillos BK, Bieniasz PD, Pornillos O. 2022. Poly(ADP-ribose) potentiates ZAP antiviral activity. PLoS Pathog 18:e1009202. 10.1371/journal.ppat.1009202. 35130321 PMC8853533

[46] Kypr J, Mrazek J, Reich J. 1989. Nucleotide composition bias and CpG dinucleotide content in the genomes of HIV and HTLV 1/2. Biochim Biophys Acta 1009:280–282. 10.1016/0167-4781(89)90114-0. 2597678

[47] Shpaer EG, Mullins JI. 1990. Selection against CpG dinucleotides in lentiviral genes: a possible role of methylation in regulation of viral expression. Nucleic Acids Res 18:5793–5797. 10.1093/nar/18.19.5793. 2170945 PMC332316

[48] Ficarelli M, Antzin-Anduetza I, Hugh-White R, Firth AE, Sertkaya H, Wilson H, Neil SJD, Schulz R, Swanson CM. 2020. CpG dinucleotides inhibit HIV-1 replication through zinc finger antiviral protein (ZAP)-dependent and -independent mechanisms. J Virol 94:e01337-19. 10.1128/JVI.01337-19. 31748389 PMC7158733

[49] Kmiec D, Nchioua R, Sherrill-Mix S, Sturzel CM, Heusinger E, Braun E, Gondim MVP, Hotter D, Sparrer KMJ, Hahn BH, Sauter D, Kirchhoff F. 2020. CpG frequency in the 5' third of the env gene determines sensitivity of primary HIV-1 strains to the zinc-finger antiviral protein. mBio 11:e02903-19. 10.1128/mBio.02903-19. 31937644 PMC6960287

[50] Nagaraj N, Wisniewski JR, Geiger T, Cox J, Kircher M, Kelso J, Paabo S, Mann M. 2011. Deep proteome and transcriptome mapping of a human cancer cell line. Mol Syst Biol 7:548. 10.1038/msb.2011.81. 22068331 PMC3261714

[51] Guttler T, Gorlich D. 2011. Ran-dependent nuclear export mediators: a structural perspective. EMBO J 30:3457–3474. 10.1038/emboj.2011.287. 21878989 PMC3181476

[52] Sun QX, Carrasco YP, Hu YC, Guo XF, Mirzaei H, MacMillan J, Chook YM. 2013. Nuclear export inhibition through covalent conjugation and hydrolysis of Leptomycin B by CRM1. Proc Natl Acad Sci USA 110:1303–1308. 10.1073/pnas.1217203110. 23297231 PMC3557022

[53] Koyama M, Shirai N, Matsuura Y. 2014. Structural insights into how Yrb2p accelerates the assembly of the Xpo1p nuclear export complex. Cell Rep 9:983–995. 10.1016/j.celrep.2014.09.052. 25437554

[54] Dong XH, Biswas A, Suel KE, Jackson LK, Martinez R, Gu HM, Chook YM. 2009. Structural basis for leucine-rich nuclear export signal recognition by CRM1. Nature 458:1136–1141. 10.1038/nature07975. 19339969 PMC3437623

[55] Monecke T, Guttler T, Neumann P, Dickmanns A, Gorlich D, Ficner R. 2009. Crystal structure of the nuclear export receptor CRM1 in complex with Snurportin1 and RanGTP. Science 324:1087–1091. 10.1126/science.1173388. 19389996

[56] Guttler T, Madl T, Neumann P, Deichsel D, Corsini L, Monecke T, Ficner R, Sattler M, Gorlich D. 2010. NES consensus redefined by structures of PKI-type and Rev-type nuclear export signals bound to CRM1. Nat Struct Mol Biol 17:1367–1376. 10.1038/nsmb.1931. 20972448

[57] Kirli K, Karaca S, Dehne HJ, Samwer M, Pan KT, Lenz C, Urlaub H, Gorlich D. 2015. A deep proteomics perspective on CRM1-mediated nuclear export and nucleocytoplasmic partitioning. Elife 4:e11466. 10.7554/eLife.11466. 26673895 PMC4764573

[58] Kudo N, Wolff B, Sekimoto T, Schreiner EP, Yoneda Y, Yanagida M, Horinouchi S, Yoshida M. 1998. Leptomycin B inhibition of signal-mediated nuclear export by direct binding to CRM1. Exp Cell Res 242:540–547. 10.1006/excr.1998.4136. 9683540

[59] Kudo N, Matsumori N, Taoka H, Fujiwara D, Schreiner EP, Wolff B, Yoshida M, Horinouchi S. 1999. Leptomycin B inactivates CRM1/exportin 1 by covalent modification at a cysteine residue in the central conserved region. Proc Natl Acad Sci USA 96:9112–9117. 10.1073/pnas.96.16.9112. 10430904 PMC17741

[60] Liu L, Chen G, Ji X, Gao G. 2004. ZAP is a CRM1-dependent nucleocytoplasmic shuttling protein. Biochem Biophys Res Commun 321:517–523. 10.1016/j.bbrc.2004.06.174. 15358138

[61] Prieto G, Fullaondo A, Rodriguez JA. 2014. Prediction of nuclear export signals using weighted regular expressions (Wregex). Bioinformatics 30:1220–1227. 10.1093/bioinformatics/btu016. 24413524

[62] Kerns JA, Emerman M, Malik HS. 2008. Positive selection and increased antiviral activity associated with the PARP-containing isoform of human zinc-finger antiviral protein. PLoS Genet 4:e21. 10.1371/journal.pgen.0040021. 18225958 PMC2213710

[63] Charron G, Li MM, MacDonald MR, Hang HC. 2013. Prenylome profiling reveals S-farnesylation is crucial for membrane targeting and antiviral activity of ZAP long-isoform. Proc Natl Acad Sci USA 110:11085–11090. 10.1073/pnas.1302564110. 23776219 PMC3703996

[64] Schwerk J, Soveg FW, Ryan AP, Thomas KR, Hatfield LD, Ozarkar S, Forero A, Kell AM, Roby JA, So L, Hyde JL, Gale M, Jr, Daugherty MD, Savan R. 2019. RNA-binding protein isoforms ZAP-S and ZAP-L have distinct antiviral and immune resolution functions. Nat Immunol 20:1610–1620. 10.1038/s41590-019-0527-6. 31740798 PMC7240801

[65] Go CD, Knight JDR, Rajasekharan A, Rathod B, Hesketh GG, Abe KT, Youn JY, Samavarchi-Tehrani P, Zhang H, Zhu LY, Popiel E, Lambert JP, Coyaud E, Cheung SWT, Rajendran D, Wong CJ, Antonicka H, Pelletier L, Palazzo AF, Shoubridge EA, Raught B, Gingras AC. 2021. A proximity-dependent biotinylation map of a human cell. Nature 595:120–124. 10.1038/s41586-021-03592-2. 34079125

[66] McDougal MB, Boys IN, De La Cruz-Rivera P, Schoggins JW. 2022. Evolution of the interferon response: lessons from ISGs of diverse mammals. Curr Opin Virol 53:101202. 10.1016/j.coviro.2022.101202. 35124511

[67] Judd EN, Gilchrist AR, Meyerson NR, Sawyer SL. 2021. Positive natural selection in primate genes of the type I interferon response. BMC Ecol Evol 21:65. 10.1186/s12862-021-01783-z. 33902453 PMC8074226

[68] Antzin-Anduetza I, Mahiet C, Granger LA, Odendall C, Swanson CM. 2017. Increasing the CpG dinucleotide abundance in the HIV-1 genomic RNA inhibits viral replication. Retrovirology 14:49. 10.1186/s12977-017-0374-1. 29121951 PMC5679385

[69] Pear WS, Miller JP, Xu L, Pui JC, Soffer B, Quackenbush RC, Pendergast AM, Bronson R, Aster JC, Scott ML, Baltimore D. 1998. Efficient and rapid induction of a chronic myelogenous leukemia-like myeloproliferative disease in mice receiving P210 bcr/abl-transduced bone marrow. Blood 92:3780–3792. 10.1182/blood.V92.10.3780. 9808572

[70] Fouchier RA, Meyer BE, Simon JH, Fischer U, Malim MH. 1997. HIV-1 infection of non-dividing cells: evidence that the amino-terminal basic region of the viral matrix protein is important for Gag processing but not for post-entry nuclear import. EMBO J 16:4531–4539. 10.1093/emboj/16.15.4531. 9303297 PMC1170079

[71] Chesebro B, Wehrly K, Nishio J, Perryman S. 1992. Macrophage-tropic human immunodeficiency virus isolates from different patients exhibit unusual V3 envelope sequence homogeneity in comparison with T-cell-tropic isolates: definition of critical amino acids involved in cell tropism. J Virol 66:6547–6554. 10.1128/JVI.66.11.6547-6554.1992. 1404602 PMC240149

[72] Derdeyn CA, Decker JM, Sfakianos JN, Wu X, O'Brien WA, Ratner L, Kappes JC, Shaw GM, Hunter E. 2000. Sensitivity of human immunodeficiency virus type 1 to the fusion inhibitor T-20 is modulated by coreceptor specificity defined by the V3 loop of gp120. J Virol 74:8358–8367. 10.1128/jvi.74.18.8358-8367.2000. 10954535 PMC116346

[73] Wei X, Decker JM, Liu H, Zhang Z, Arani RB, Kilby JM, Saag MS, Wu X, Shaw GM, Kappes JC. 2002. Emergence of resistant human immunodeficiency virus type 1 in patients receiving fusion inhibitor (T-20) monotherapy. Antimicrob Agents Chemother 46:1896–1905. 10.1128/AAC.46.6.1896-1905.2002. 12019106 PMC127242

[74] Platt EJ, Wehrly K, Kuhmann SE, Chesebro B, Kabat D. 1998. Effects of CCR5 and CD4 cell surface concentrations on infections by macrophagetropic isolates of human immunodeficiency virus type 1. J Virol 72:2855–2864. 10.1128/JVI.72.4.2855-2864.1998. 9525605 PMC109730

[75] Sievers F, Higgins DG. 2014. Clustal Omega, accurate alignment of very large numbers of sequences. Methods Mol Biol 1079:105–116. 10.1007/978-1-62703-646-7_6. 24170397

[76] Deng W, Maust BS, Nickle DC, Learn GH, Liu Y, Heath L, Kosakovsky Pond SL, Mullins JI. 2010. DIVEIN: a web server to analyze phylogenies, sequence divergence, diversity, and informative sites. Biotechniques 48:405–408. 10.2144/000113370. 20569214 PMC3133969

[77] Letunic I, Bork P. 2021. Interactive Tree Of Life (iTOL) v5: an online tool for phylogenetic tree display and annotation. Nucleic Acids Res 49:W293–W296. 10.1093/nar/gkab301. 33885785 PMC8265157

[78] Ashkenazy H, Erez E, Martz E, Pupko T, Ben-Tal N. 2010. ConSurf 2010: calculating evolutionary conservation in sequence and structure of proteins and nucleic acids. Nucleic Acids Res 38:W529–533. 10.1093/nar/gkq399. 20478830 PMC2896094

[79] Edgar RC. 2004. MUSCLE: a multiple sequence alignment method with reduced time and space complexity. BMC Bioinformatics 5:113. 10.1186/1471-2105-5-113. 15318951 PMC517706

