# Supplementary material for: A Nuclear Export Signal in KHNYN Required for Its Antiviral Activity Evolved as ZAP Emerged in Tetrapods
Source: J Virol. 2023 Jan 12;97(1):e00872-22. doi: 10.1128/jvi.00872-22 (PMC9888277; doi:10.1128/jvi.00872-22)
Supplement: Supplemental file 1 — Fig. S1 to S3. Download jvi.00872-22-s0001.pdf, PDF file, 0.2 MB [file jvi.00872-22-s0001.pdf]

```
Homo sapiens KHNYN    1                           MPTWGARPA----SPDRFAVSAEAE  21
Mus musculus KHNYN    1                           MSTWGFASP----TPDRFAVSAEAE  21
Danio rerio KHNYN     1                           MSLSVDECGPQEQVEDEFTCAGVLR  25
Homo sapiens N4BP1    1                              MAARA-----VLDEFTAPAEKA  17
Mus musculus N4BP1    1                              MAARV-----VLDEFTAPAEKA  17
Danio rerio N4BP1     1 MSTTRPLLGMKRITEVTCTEPPGGRQSPTASRAQPDSLTVDEFTVHEDKQ  50
                                                                * *
```

Extended di-KH domain

```
Homo sapiens KHNYN   22 NKVREQQPHVERIFSVGVSVL------PKDCPDNPH-IWLQLEGPKENAS  64
Mus musculus KHNYN   22 DKVREQQTRLERIFNVGMSVL------SKDCPENPH-IWLQLEGPKENVC  64
Danio rerio KHNYN    26 EAIRALQPTVERVFGVKLSIG------AEESPSQGGQIWLQLRGARTQVT  69
Homo sapiens N4BP1   18 ELLEQSRGRIEGLFGVSLAVLGAL---GAEEPLPAR-IWLQLCGAQEAVH  63
Mus musculus N4BP1   18 ALLERSRGRIEALFGVGLAVLGAL---GAEEPLPAR-IWLQLRGAQEAVH  63
Danio rerio N4BP1    51 TELKCSKPKVEQVFQVTFTIIGLLDHTGAHGSKASRQIWLQLKGKKEDVY 100
                                  *  * *                     ***** *
```

Extended di-KH domain

```
Homo sapiens KHNYN   65 RAKEYLKGLCSPELQDEIHYPPKLHCIFLGAQGFFLDCLAWSTSAHLVPR 114
Mus musculus KHNYN   65 RAKEYLKGLCSPELQSEIHYPPRLHCIFLGAHGFFLDCLAWSTSAHLVPL 114
Danio rerio KHNYN    70 AAKLFVKGVVNQEAQKEMQFPEVLHCIFCGAKGLFMDCLIKHTSAHMVVG 119
Homo sapiens N4BP1   64 SAKEYIKGICEPELEERECYPKDMHCIFVGAESLFLKSLIQDTCADLCIL 113
Mus musculus N4BP1   64 SAKEYIKGICEPELEEKECYPKAMHCIFVGAQSLFLKSLIQDTCADLCVL 113
Danio rerio N4BP1   101 KAKEYVKGLCDPELQKEEWYPVDMHCIFAGARGLFLDRLLRDTSAEVQVL 150
                         **   **    *       *   **** **   *   *   * *
```

Extended di-KH domain

```
Homo sapiens KHNYN 115 APGSLMISGLTEAFVMAQSRVEELAERLSWDFTPGPSSGASQCTGVLRDF 164
Mus musculus KHNYN 115 LPGSLMISGLTEAFVMAQSRVEELVQRLSWDLQLQSCPGAPDNGGVLRDF 164
Danio rerio KHNYN  120 SPGFLLISGLTEPVVKAFSFIVDLVEKYRSGQGRRPDSAG-ASLESRRAF 168
Homo sapiens N4BP1 114 DIGLLGIRGSAEAVVMARSHIQQFVKLFE---NKENLPSSQKESEVKREF 160
Mus musculus N4BP1 114 DTGLLGIRGSAEAVVMARSHIQQFVKLFE---SNENLPSNQRESEIKREF 160
Danio rerio N4BP1  151 EPGRLKLSGCAEAVVMAQSRVQQFVALFQ---EKRSLPAD-REPSVKRKF 196
                         * *   *  *  * * *                            * *
```

Extended di-KH domain

```
Homo sapiens KHNYN 165 SALLQSPGDAHREALLQLPLAVQEELLSLVQ------------------- 195
Mus musculus KHNYN 165 SALLQTREDAYTEALLRLPLAVQEELLSLVQ------------------- 195
Danio rerio KHNYN  169 KTLVEELEDRHTLELLALPVRVKEALLELVR------------------- 199
Homo sapiens N4BP1 161 KQFVEAHADNYTMDLLILPTSLKKELLTLTQGEENLFETGDDEVIEMRDS 210
Mus musculus N4BP1 161 RQFVEAHADSYTMDLLILPTSLKKELLSLTQGEESLFET-DDDVITVGDV 209
Danio rerio N4BP1  197 KTFVEDRADKYAMELLLLPSALKEELLGLAQS--------PTQPIVIIDL 238
                               *     ** **      ** *
```

```
Homo sapiens KHNYN 196 -----------------------EASSGQGPGALASWEGR---------- 212
Mus musculus KHNYN 196 -----------------------EASRGQGPSREVG-------------- 208
Danio rerio KHNYN  200 -----------------------LAGNTHTRTLQNPENRH---------- 216
Homo sapiens N4BP1 211 QQTEFTQNAATGLNISRDETVLQEEARNKAGTPVSELTKQ-MDTVLSSSP 259
Mus musculus N4BP1 210 RPPEYTQSAATGPSSARDEVVVQEDSRNKARTPVSELTKH-MDTVFSSSP 258
Danio rerio N4BP1  239 -----------------------EQDRSQTSTPVTDLSNRILDTTFE--- 262
```

```
Homo sapiens KHNYN 213 --------------SSALLGAQCQGVRAPPSDGRE-----SLDTGSMGPG 243
Mus musculus KHNYN 209 --------------SSGLLSPQFQGVRAPLNEGRE-----FVGTRVAGSG 239
Danio rerio KHNYN  217 --------------THTLQDAATTLQENGGVHEAS-------DNNNSAES 245
Homo sapiens N4BP1 260 DVLFDPINGLTPDEEALSNERICQKRRFSDSEERHTKKQFSLENVQEGEI 309
Mus musculus N4BP1 259 DVLFVPVNGLSPDEDALSKDRVCHKRRSSDTEERHTKKQFSLENVPEGEL 308
Danio rerio N4BP1  263 ----DKTSPITPEVMPGLNGRPCNKRRSSESEQRDTKRQYSLERREEEQC 308

                                                          UBA-like domain

Homo sapiens KHNYN 244 DCRGARGDTYAVEKEGGKQGGPREMDWGWKELPGEEAWE------REVAL 287
Mus musculus KHNYN 240 KSPAVRGQSHTVEKEERKQDAVRDMGSGRKELSGEEVWE------PGVAY 283
Danio rerio KHNYN  246 SHSQRPLLGFTLDSRHFQTPEPPQPDRPPESSGCGEEFQHLLKFFTAMGF 295
Homo sapiens N4BP1 310 LHDAKTLAGNVIADLSDSSADSENLSPDIKETTEEMEYNILVNFFKTMGY 359
Mus musculus N4BP1 309 LPDGKGSAGNEVIDLSDPASNSTNLSPDGKDTTEEMEYNILVNFFKTMGY 358
Danio rerio N4BP1  309 EEREREPTKTWTVKSAKGTLAASEMTNESEAVSPETNLRCLVNFFRTMGY 358

                                   UBA-like domain

Homo sapiens KHNYN 288 RPQSVGGGARESAPLKGKALGKEEIALGGGGFCVHRE-PPGAHGSCHRAA 336
Mus musculus KHNYN 284 RSQLAGGGAEEVAPLKGKASGKQEVPQQRGGFSVQGE-PSGAHVPCQRAA 332
Danio rerio KHNYN  296 TEAVVRSVLARTGP-KEASQLLDLIQQEQDKTDQQNQLGSGEMHAVERPE 344
Homo sapiens N4BP1 360 SQEIVEKVIKVYGPSTEPLLLLEEIEKENKRFQEDREFSAGTVYPETNKT 409
Mus musculus N4BP1 359 SQEIVEKVIREYGPSTEPLLLLEEIEKENKRLQEDRDFPPCTVYPDASQS 408
Danio rerio N4BP1  359 QQDVVERVVRETGQTEDTFLLLERIVEETQKTQSTQGAQRTSRTPDPSPC 408

Homo sapiens KHNYN 337 QSRGASLLQR--------------------LHNGNASPPRVPSPPPAPE- 365
Mus musculus KHNYN 333 PIRGASLLQR--------------------LHNGSASPPRVPSPPPAPE- 361
Danio rerio KHNYN  345 ANQTDAKQDDFVLGVLKAAAATCGYTEEHVMEVYGNLPEIKPHELLMQLQ 394
Homo sapiens N4BP1 410 KNKGVYSSTN-ELTTDSTPKKTQAHTQQNMVEKFSQLPFKVEAKPCTSN- 457
Mus musculus N4BP1 409 RNAGVGSTTN-ELTADSTPKKAQSHTEQSMVERFSQLPFK-DSKHCTSN- 455
Danio rerio N4BP1  409 ANASSTSTSN-RLKEK----------ERVQMRALAEIKCKENIRPPSTNG 447
```

```
Homo sapiens KHNYN 366 -----------------PPWHCGDRGDCG-DRGDVGDRGDKQQGMAR--- 394
Mus musculus KHNYN 362 -----------------PPWPCGDRDRDR----DRGDRGDKQQAGAR--- 387
Danio rerio KHNYN  395 KQEHAHFNGLRNGSQQ-ADWTTEQNSRSYVENLDPGSRNRASNHESS--- 440
Homo sapiens N4BP1 458 -CRINTFRTVPIEQKH-EVWGSNQNYICNTDPETDGLSPSVASPSPK--- 502
Mus musculus N4BP1 456 -CKVNSFRTVPVGQKQ-EIWGSKQNSSCTVDLETDGHSASAASASPK--- 500
Danio rerio N4BP1  448 IGQKNQTSSVPLASATLKRNNGAQTDLCEVIIIDDEEDFTETERKPRLTP 497

Homo sapiens KHNYN 395 ------------GRGP----------QWK--------------------- 401
Mus musculus KHNYN 388 ------------GRGS----------PWK--------------------- 394
Danio rerio KHNYN  441 ------KPVSIPGSGSTVKGPPQMTYSWENMTSDIH--PVNSYSQSP--- 479
Homo sapiens N4BP1 503 -------EVNFVSRGASSHQPRVPLFPENGLHQQPE--PLLPNNMKSACE 543
Mus musculus N4BP1 501 -------DISFVSRGASGHQQRNPAFPENGFQQQTE--PLLPNNTKPACE 541
Danio rerio N4BP1  498 LDLKPESRFDYLPRGSSQTMVPVRMETVTNLRSSSQGPPLRTSDTRPGC- 546
                                      *

Homo sapiens KHNYN 402 ------------------------------------RGARG--GNLVTGT 413
Mus musculus KHNYN 395 ------------------------------------RGTRG--GNLVTGT 406
Danio rerio KHNYN  480 ---------------PNVKQSLDVTSAKFSNTIPKAKPERGAVASVVTGP 514
Homo sapiens N4BP1 544 KRLGCCSSPHSKPNCSTLSPPMPLPQLLPSVTDARSAGPSDHIDSSVTGV 593
Mus musculus N4BP1 542 KRSGSCSSPQPKPNYPPLSPPLPLPQLLPSVTEARLGGSSDHIDSSVTGV 591
Danio rerio N4BP1  547 -------------SYQTLPGRAPLP---------RSEAQYTSKAAPLTGM 574
                                                                      **
```

PIN domain

```
Homo sapiens KHNYN 414 QRFKEALQDPFTLCLANVPGQPDLRHIVIDGSNVAMVHGLQHYFSSRGIA 463
Mus musculus KHNYN 407 QRFQEALQDPFTLCLANVPGQPDLRHIVIDGSNVAMVHGLQHYFSSRGIA 456
Danio rerio KHNYN  515 QRFLEGLKKPFSLQLSDQPGDAQLRHVIIDGSNVAMSHGLGVFFSCRGIA 564
Homo sapiens N4BP1 594 QRFRDTLKIPYKLELKNEPGRTDLKHIVIDGSNVAITHGLKKFFSCRGIA 643
Mus musculus N4BP1 592 QRFRDTLKIPYKLELKNEPGRADLKHIVIDGSNVAITHGLKKFFSCRGIA 641
Danio rerio N4BP1  575 SRFQQSLRTPYRLILQNEPGSPNLRHIIIDGSNVAMAHGLHRVFSCRGIA 624
                        **   *  *  * *   **   * *  *******  ***   ** ****
```

PIN domain

```
Homo sapiens KHNYN 464 IAVQYFWDRGHRDITVFVPQWRFSKDAKVRESHFLQKLYSLSLLSLTPSR 513
Mus musculus KHNYN 457 LAVQYFWDRGHRDITVFVPQWRFSKDSKVRESHFLQKLYSLSLLSLTPSR 506
Danio rerio KHNYN  565 LAVQHFWAEGHREIMVFVPQWRQKNNSKIKEKHYLNELHDLGLLSYTPSR 614
Homo sapiens N4BP1 644 IAVEYFWKLGNRNITVFVPQWRTRRDPNVTEQHFLTQLQELGILSLTPAR 693
Mus musculus N4BP1 642 IAVEYFWKLGNRNITVFVPQWRTRRDPNITEQHFLTQLQELGILSLTPAR 691
Danio rerio N4BP1  625 IAVEAFWRRGHREITVFVPQWRQKKDPNITEQHFLNQLENLRLLSFTPSR 674
                        **  **  * * * *******        * * *  *  *  ** ** *
```

PIN domain

```
Homo sapiens KHNYN 514 VMDGKRISSYDDRFMVKLAEETDGIIVSNDQFRDLAEESEKWMAIIRERL 563
Mus musculus KHNYN 507 VMDGKRISSYDDRFMVKLAEETDGIIVSNDQFRDLAEESDKWMAIIRERL 556
Danio rerio KHNYN  615 EVEGKRIISYDDRFMLDLAQKTNGVIVTNDNLRDLVDESPAWRDIIKKSL 664
Homo sapiens N4BP1 694 MVFGERIASHDDRFLLHLADKTGGIIVTNDNFREFVNESVSWREIITKRL 743
Mus musculus N4BP1 692 MVFGERIASHDDRFLLHLADKTGGIIVTNDNFREFVTESVSWREIITKRL 741
Danio rerio N4BP1  675 EVCGHRISSHDDRFLLHLAEKTGGVIVTNDNLRDFVSQSEAWRRIIHERL 724
                          * ** * ****   **  * * ** **  *     *  *  **   *
```

PIN domain

```
Homo sapiens KHNYN 564 LPFTFVGNLFMVPDDPLGRNGPTLDEFLKKPA------------------ 595
Mus musculus KHNYN 557 LPFTFVGNLFMVPDDPLGRNGPTLDEFLKKPV------------------ 588
Danio rerio KHNYN  665 LQYVFAGDQFMLPDDPLGRGGPHLRDFLHKHN-------SSSPVPSSHSF 707
Homo sapiens N4BP1 744 LQYTFVGDIFMVPDDPLGRSGPRLEEFLQKEVCLRDMQPLLSALPNVGMF 793
Mus musculus N4BP1 742 LQYTFVGDIFMVPDDPLGRNGPRLEEFLRKEAFLRHMQPLLNALPSVGTF 791
Danio rerio N4BP1  725 LQFTFVEDHFMIPDDPLGKHGPHLDEFLLKDS------RGSPIIPPLRTD 768
                       *   *    ** ******  ** *  ** *
```

```
Homo sapiens KHNYN 596 -------RTQGSSKAQHP--------SRGFAEHGKQQQGR------EEEK 624
Mus musculus KHNYN 589 -------RKQGSSKTQQP--------SKGSTEQANQQQGK------DADR 617
Danio rerio KHNYN  708 -----AGVSSPSSAAPAPRAHTDGLQYRNWT-PGSHGWGQG-----SSAG 746
Homo sapiens N4BP1 794 DPSFRVPGTQAASTSHQPPTRIQGAPSSHWLPQQPHFPLLPALPSLQQNL 843
Mus musculus N4BP1 792 DPGFRSPSTQVANNSHQPPPRIQ-TSSSPWLPQQSHFTALATLPSMQQNP 840
Danio rerio N4BP1  769 LRATPSVYSQAAQSTAHP------SSPSHWPHSGPPDWHLP-----RPSP 807
                                        *
```

CUBAN/CoCUN domain

```
Homo sapiens KHNYN 625 GSGGIRKTRETERLRRQLLEVFWGQDHK--VDFILQREPYCRDINQLSEA 672
Mus musculus KHNYN 618 SNGGIRKTRETERLRRQLLEVFWGQDHK--VDFILQREPYCRDINQLSEA 665
Danio rerio KHNYN  747 EEVNERSLEETRRLRQSLVSIFPGQESV--IIMILQCHPNIRDINRLTEL 794
Homo sapiens N4BP1 844 PMPAQRSSAETNELREALLKIFPDSEQRLKIDQILVAHPYMKDLNALSAM 893
Mus musculus N4BP1 841 PLPAQRSSAETSELREALLKIFPDSEQKLKIDQILAAHPYMKDLNALSAL 890
Danio rerio N4BP1  808 SPPPQRSPSETTELKRKLYDIFPDQKQR--IDRILSDNPYMRDLNALSGL 855
                            *   **  *   *   *           **   *   * * *
```

```
Homo sapiens KHNYN 673 LLSLNF 678
Mus musculus KHNYN 666 LLSLNF 671
Danio rerio KHNYN  795 ILEQQE 800
Homo sapiens N4BP1 894 VLD    896
Mus musculus N4BP1 891 VLD    893
Danio rerio N4BP1  856 LLG    858
                        *
```

Fig. S1: Regions in human, mouse and zebrafish KHNYN and N4BP1 with predicted structure by AlphaFold. MUSCLE alignment of KHNYN and N4BP1 orthologs from human, mouse and zebrafish. The extended di-KH, UBA-like, PIN and CUBAN or CoCUN domains are marked in the alignment. Residues with a AlphaFold per-residue confidence score (pLDDT) 90>pLDDT>70 are highlighted in yellow and pLDDT>90 are highlighted in green. * indicates residues that are identical in all three KHNYN and N4BP1 orthologs.

A

```
                                 Extended di-KH domain

Human KHNYN    1 MPTWGARPASPDRFAVSAEAENKVREQQPHVERIFSVGVSVLPKDCPDNP---HIWLQLE  57
Human N4BP1    1    MAAR-AVLDEFTAPAEKAELLEQSRGRIEGLFGVSLAVLGALGAEEPLPARIWLQLC  56
                      ** *  * *.  **    . . . ..* .* * ..**     . *   .*****

                                 Extended di-KH domain

Human KHNYN   58 GPKENASRAKEYLKGLCSPELQDEIHYPPKLHCIFLGAQGFFLDCLAWSTSAHLVPRAPG 117
Human N4BP1   57 GAQEAVHSAKEYIKGICEPELEERECYPKDMHCIFVGAESLFLKSLIQDTCADLCILDIG 116
                 * .*    ****.**.* ***..   **  .****.**.  ** .*   *.* *     *

                                 Extended di-KH domain

Human KHNYN  118 SLMISGLTEAFVMAQSRVEELAERLSWDFTPGPSSGASQCTGVLRDFSALLQSPGDAHRE 177
Human N4BP1  117 LLGIRGSAEAVVMARSHIQQFVKLFE---NKENLPSSQKESEVKREFKQFVEAHADNYTM 173
                  * * *.** ***.*....         .      . . . * *.*   ...  *

Human KHNYN  178 ALLQLPLAVQEELLSL------------------------------------------VQ 195
Human N4BP1  174 DLLILPTSLKKELLTLTQGEENLFETGDDEVIEMRDSQQTEFTQNAATGLNISRDETVLQ 233
                  ** ** ... ***.*                                          .*

Human KHNYN  196 EASSGQGPGALASWEGRSSALLGAQ-----------------------CQGVRAPPSDGR 232
Human N4BP1  234 EEARNKAGTPVSELTKQMDTVLSSSPDVLFDPINGLTPDEEALSNERICQKRRFSDSEER 293
                 * . ..    ..    .  ..* .                        **  *   *. *

Human KHNYN  233 --------ESLDTGSMGPGDCRGARGDTYAVEKEGGKQGG---------PREMDW----- 270
Human N4BP1  294 HTKKQFSLENVQEGEI-LHDAKTLAGNVIADLSDSSADSENLSPDIKETTEEMEYNILVN 352
                         * .  * .   *..   *   *   .                 **.
```

UBA-like domain

```
Human KHNYN  271 -----GW------------------------------------KELPGEEAWEREVALRPQ 290
Human N4BP1  353 FFKTMGYSQEIVEKVIKVYGPSTEPLLLLEEIEKENKRFQEDREFSAGTVYPETNKTKNK 412
                      *                                    .*             . .

Human KHNYN  291 SVGGGARE----SAPLKGKALGKEEI---------------------------------- 312
Human N4BP1  413 GVYSSTNELTTDSTPKKTQAHTQQNMVEKFSQLPFKVEAKPCTSNCRINTFRTVPIEQKH 472
                  *   . *    *.* * .*  .. .

Human KHNYN  313 -ALGGGGFCVHREPPGAHGSCHRAAQSRGASLLQRLHNGNASPPRVPSPP-----PAPEP 366
Human N4BP1  473 EVWGSNQNYICNTDPETDGLSPSVASPSPKEVNFVSRGASSHQPRVPLFPENGLHQQPEP 532
                    * .   .    * . * .   *      .    ..  .  ****  *       ***

Human KHNYN  367 PWHCGDRGDCGDRGDVGDRGDKQQGM------------------ARGRGPQWKRGARGGN 408
Human N4BP1  533 LLPNNMKSACEKRLGCCSSPHSKPNCSTLSPPMPLPQLLPSVTDARSAGP----SDHIDS 588
                     . .  *  *         . .                   **  **      .
```

PIN domain

```
Human KHNYN  409 LVTGTQRFKEALQDPFTLCLANVPGQPDLRHIVIDGSNVAMVHGLQHYFSSRGIAIAVQY 468
Human N4BP1  589 SVTGVQRFRDTLKIPYKLELKNEPGRTDLKHIVIDGSNVAITHGLKKFFSCRGIAIAVEY 648
                  *** ***...*. *. * * * **. **.**********. ***...**.*******.*
```

PIN domain

```
Human KHNYN  469 FWDRGHRDITVFVPQWRFSKDAKVRESHFLQKLYSLSLLSLTPSRVMDGKRISSYDDRFM 528
Human N4BP1  649 FWKLGNRNITVFVPQWRTRRDPNVTEQHFLTQLQELGILSLTPARMVFGERIASHDDRFL 708
                 **  *.* *********  .*  * * *** .*  * .*****.*.. * **.* ****.
```

PIN domain

```
Human KHNYN  529 VKLAEETDGIIVSNDQFRDLAEESEKWMAIIRERLLPFTFVGNLFMVPDDPLGRNGPTLD 588
Human N4BP1  709 LHLADKTGGIIVTNDNFREFVNESVSWREIITKRLLQYTFVGDIFMVPDDPLGRSGPRLE 768
                 ..**. * ****.**.**.   **  *  **  *** .**** .********** ** *.

Human KHNYN  589 EFLKK--------------------------PARTQGSSKAQHPSR---GFAEHGKQQQG 619
Human N4BP1  769 EFLQKEVCLRDMQPLLSALPNVGMFDPSFRVPGTQAASTSHQPPTRIQGAPSSHWLPQQP 828
                 ***.*                          *     *.  * *.*     . *   **
```

CUBAN/CoCUN domain

```
Human KHNYN  620 R----------EEEKGSGGIRKTRETERLRRQLLEVFWGQDH--KVDFILQREPYCRDIN 667
Human N4BP1  829 HFPLLPALPSLQQNLPMPAQRSSAETNELREALLKIFPDSEQRLKIDQILVAHPYMKDLN 888
                 .          ..       * . **  **  ** .*   ..  *.* **   ** .*.*

Human KHNYN  668 QLSEALLSLNF 678
Human N4BP1  889 ALSAMVLD    896
                  **  .*
```

B

```
Zebrafish KHNYN   1                          MSLSVDECGPQEQVEDEFTCAGVLREAIRALQPTV  35
Zebrafish N4BP1   1 MSTTRPLLGMKRITEVTCTEPPGGRQSPTASRAQPDSLTVDEFTVHEDKQTELKCSKPKV  60
                                              * .   . *     ****     .  ... .* *
```

Extended di-KH domain

```
Zebrafish KHNYN  36 ERVFGVKLS-IGAEESPSQGG-----QIWLQLRGARTQVTAAKLFVKGVVNQEAQKEMQF  89
Zebrafish N4BP1  61 EQVFQVTFTIIGLLDHTGAHGSKASRQIWLQLKGKKEDVYKAKEYVKGLCDPELQKEEWY 120
                    *.** *  . **  .     *     ******.* .  *  ** .***.   * ***  .
```

Extended di-KH domain

```
Zebrafish KHNYN  90 PEVLHCIFCGAKGLFMDCLIKHTSAHMVVGSPGFLLISGLTEPVVKAFSFIVDLVEKYRS 149
Zebrafish N4BP1 121 PVDMHCIFAGARGLFLDRLLRDTSAEVQVLEPGRLKLSGCAEAVVMAQSRVQQFVALF-- 178
                    *  .****.**.***.* *.. *** . *  ** * .** .* ** * * .   *  .
```

Extended di-KH domain

```
Zebrafish KHNYN 150 GQGRRPDSAGASLESRRAFKTLVEELEDRHTLELLALPVRVKEALLELV----------- 198
Zebrafish N4BP1 179 -QEKRSLPADREPSVKRKFKTFVEDRADKYAMELLLLPSALKEELLGLAQSPTQPIVIID 237
                     * .*   *      .* *** **.  *. ..*** **  .** ** *

Zebrafish KHNYN 199 ----RLAGNTHTRTLQNPENRHTHTLQDAATTLQENGGVHEASDNNNSAESSHSQRPLLG 254
Zebrafish N4BP1 238 LEQDRSQTSTPVTDLSNRILDTTFEDKTSPITPEVMPGLNGRPCNKRRSSESEQRDTKRQ 297
                        *    *    * *     *   . .  * .   *..    *   .  * .

Zebrafish KHNYN 255 FTLDSRHFQTPEPPQPDRPP--------------------ESSGCGEE--FQHLLKFFTA 292
Zebrafish N4BP1 298 YSLERR--EEEQCEEREREPTKTWTVKSAKGTLAASEMTNESEAVSPETNLRCLVNFFRT 355
                    ..*. *  .  .  . .* *                    **     *   . *. ** .
```

UBA-like domain

```
Zebrafish KHNYN 293 MGFTEAVVRSVLARTGPKEAS-QLLDLIQQEQDKTDQQNQLGSGEMHAVERPEANQTDAK 351
Zebrafish N4BP1 356 MGYQQDVVERVVRETGQTEDTFLLLERIVEETQKTQSTQGAQRTSRTPDPSPCANASSTS 415
                    **. . **  *.  **  * .  **. * .*  **   .            * ** . .

Zebrafish KHNYN 352 QDDFV-----LGVLKAAAATCGYTEEHVMEVYGNLPEIKPHELLMQLQKQEHAHFNGLRN 406
Zebrafish N4BP1 416 TSNRLKEKERVQMRALAEIKC---KENIRPPSTNGIGQKNQTSSVPLASATLKRNNGAQT 472
                        .     . .   *   *    *..     *    * .   . *      . ** ..

Zebrafish KHNYN 407 G-------SQQADWT-TEQNSRSYVENLDPGSRNRASNHESSKPV------SIPGSGSTV 452
Zebrafish N4BP1 473 DLCEVIIIDDEEDFTETERKPRLTPLDLKPESRFDYLPRGSSQTMVPVRMETVTNLRSSS 532
                              . * * **.  *     * * **     . **. .      .. .  *.

Zebrafish KHNYN 453 KGPPQMTYSWENMTSDIHPVNSYSQSPPNVKQSLDVTSAKFSNTIPKAKPERGAVASVVT 512
Zebrafish N4BP1 533 QGPPL-------RTSDTRPGCSYQTLPGRA-------------PLPRSEAQYTSKAAPLT 572
                    .***         *** .*  **   *                 .*..  .  . *. .*
```

PIN domain

```
Zebrafish KHNYN 513 GPQRFLEGLKKPFSLQLSDQPGDAQLRHVIIDGSNVAMSHGLGVFFSCRGIALAVQHFWA 572
Zebrafish N4BP1 573 GMSRFQQSLRTPYRLILQNEPGSPNLRHIIIDGSNVAMAHGLHRVFSCRGIAIAVEAFWR 632
                    *  ** . *. *. * *  .**  .***.*********.***   *******.**. **
```

PIN domain

```
Zebrafish KHNYN 573 EGHREIMVFVPQWRQKNNSKIKEKHYLNELHDLGLLSYTPSREVEGKRIISYDDRFMLDL 632
Zebrafish N4BP1 633 RGHREITVFVPQWRQKKDPNITEQHFLNQLENLRLLSFTPSREVCGHRISSHDDRFLLHL 692
                     ***** *********    * *.*.**.*  * ***.****** *.** * ****.* *
```

PIN domain

```
Zebrafish KHNYN 633 AQKTNGVIVTNDNLRDLVDESPAWRDIIKKSLLQYVFAGDQFMLPDDPLGRGGPHLRDFL 692
Zebrafish N4BP1 693 AEKTGGVIVTNDNLRDFVSQSEAWRRIIHERLLQFTFVEDHFMIPDDPLGKHGPHLDEFL 752
                    *.**.*********** * .* *** **.  ***. *  *.**.******. **** .**

Zebrafish KHNYN 693 HKHNSSSPVPSSHSFAGVSSPSSAAPAPRAHTDGLQYRNW-TPGSHGWGQGSSAGEEVNE 751
Zebrafish N4BP1 753 LKDSRGSPIIPPLRTDLRATPSVYSQAAQSTAHPSSPSHWPHSGPPDWHLPRPSPSPPPQ 812
                     *    **.         ..**  . * .. .      .*   *   *     .     .
```

CUBAN/CoCUN domain

```
Zebrafish KHNYN 752 RSLEETRRLRQSLVSIFPGQESVIIMILQCHPNIRDINRLTELILEQQE 800
Zebrafish N4BP1 813 RSPSETTELKRKLYDIFPDQKQRIDRILSDNPYMRDLNALSGLLLG    858
                    **  **  *.. *  *** *   *  **  .* .**.* *. *.*
```

C

Alignment	**Overall protein** (identity / similarity)	**di-KH domain** (identity / similarity)	**UBA-like** (identity / similarity)	**PIN domain** (identity / similarity)	**CUBAN domain** (identity / similarity)
**Human KHNYN and human N4BP1**	28% / 10%	34% / 12%	N/A	61% / 10%	39% / 16%
**Zebrafish KHNYN and zebrafish N4BP1**	34% / 9%	40% / 13%	34% / 10%	63% / 8%	42% / 8%

Fig. S2: KHNYN and N4BP1 are paralogs that have homology in the structural domains. MUSCLE alignment of human **(A)** or zebrafish **(B)** KHNYN and N4BP1 paralogs. The extended di-KH, UBA-like, PIN and CUBAN/CoCUN domains are marked in the alignment. "*" indicates residues that are identical in KHNYN and N4BP1. "." indicates residues that are similar in KHNYN and N4BP1. (**C**) The % amino acid identity and similarity in the overall protein and each domain between the KHNYN and N4BP1 paralogs.

```
Homo sapiens KHNYN                  630 RKTRETERLRRQLLEVFWG--QDHKVDFILQREPYCRDINQLSEALLSLNF      678
Mus musculus KHNYN                  623 RKTRETERLRRQLLEVFWG--QDHKVDFILQREPYCRDINQLSEALLSLNF      671
Danio rerio KHNYN                   752 RSLEETRRLRQSLVSIFPG--QESVIIMILQCHPNIRDINRLTELILEQQE      800
Homo sapiens N4BP1                  849 RSSAETNELREALLKIFPDSEQRLKIDQILVAHPYMKDLNALSAMVLD         896
Mus musculus N4BP1                  846 RSSAETSELREALLKIFPDSEQKLKIDQILAAHPYMKDLNALSALVLD         893
Danio rerio N4BP1                   813 RSPSETTELKRKLYDIFPD--QKQRIDRILSDNPYMRDLNALSGLLLG         858
Amblyraja radiata N4BP1-like        808 RPKEETERLKQDLFKIFPDAPQRERIDRILAAHPLMRDPNALSAMVLDQE       857
Callorhinchus milii N4BP1-like      839 RSVEETERLKEELLKIFPDFKQRQKIDQILRAHPYMRDLNALSAMVLDQE       888
Carcharodon carcharias N4BP1-like   813 RSKEETEQLKQELLKIFPEFRQRQKIDQILAAHPHMRDPNALSAMVLDQEEIAM   866
Chiloscyllium plagiosum N4BP1-like  807 RTREETDQLKQNLLKIFPEVSQRQKIDQILAAHPHMRDPNALSAMVLDQE       856
Scyliorhinus canicula N4BP1-like    810 RSKEETEQLKQELLKIFPEFRQRQKIDRILAAHPHMRDPNALSAMVLDQEEIAL   863
Petromyzon marinus KHNYN-like       808 KPRGEVGGGRGGLLAVFPAS--AAQVEAVLRGNPGLTDVAELADLVLPLI       855
Petromyzon marinus N4BP1-like       924 RPTNDLQDLRRQLLDIFPAD--EEKVQRVLQQYPAVKDLNLLSDLMLDV        970
Branchiostoma belcheri N4BP1-like   938 ISQAQV-QLFQQLMQVFPG--QEDKIRRVLQSNPEMADLNAVSAMVLDVDL      985
Acanthaster planci N4BP1-like       904 RKDAKDHPCYEMLMGIFPN--NEEEVLRVLTKNSDKEDANELVIQVLVEKEKSE   955
Aplysia californica N4BP1-like      850 RSEEETQRLFKQLSQVFCDPSQSSCIKKVLQNHKDETDINRLTNFLLSAMDL     901
Biomphalaria glabrata N4BP1-like    640 RSREETQRLYEQLIQVFPNKDQSLRVYQVLENHCTETDLVKLTNYVMNALFSKDH  694
Pomacea canaliculata N4BP1-like     643 RPPDVTESLFMALKQVFPNEDQDYKIRAVLDNHETETDLNRLTNYCMSALFL     694
                                                    *   *            *       *
```

Fig. S3. The CUBAN domain is conserved in cartilaginous fish, lamprey, lancelet, echinoderm and mollusc N4BP1-like proteins. MUSCLE alignment of CUBAN domains from KHNYN, N4BP1 or N4BP1-like proteins from the indicated species. Residues highlighted are highly conserved in the KHNYN CUBAN domain (ratio >0.85) in Figure 5A. *Amblyraja radiata*, *Callorhinchus milii*, *Carcharodon carcharias*, *Chiloscyllium plagiosum* and *Scyliorhinus canicular* are cartilaginous fishes. *Petromyzon marinus* Is a lamprey. *Branchiostoma belcheri* Is a lancelet. *Acanthaster planci* is an echinoderm. *Aplysia californica*, *Biomphalaria glabrata* and *Pomacea canaliculate* are molluscs.
